# Complete genome sequence of the extremely acidophilic methanotroph isolate V4, *Methylacidiphilum infernorum*, a representative of the bacterial phylum *Verrucomicrobia*

**DOI:** 10.1186/1745-6150-3-26

**Published:** 2008-07-01

**Authors:** Shaobin Hou, Kira S Makarova, Jimmy HW Saw, Pavel Senin, Benjamin V Ly, Zhemin Zhou, Yan Ren, Jianmei Wang, Michael Y Galperin, Marina V Omelchenko, Yuri I Wolf, Natalya Yutin, Eugene V Koonin, Matthew B Stott, Bruce W Mountain, Michelle A Crowe, Angela V Smirnova, Peter F Dunfield, Lu Feng, Lei Wang, Maqsudul Alam

**Affiliations:** 1 Advance Studies in Genomics, Proteomics and Bioinformatics, College of Natural Sciences, University of Hawaii, Keller Hall #319, Honolulu, Hawaii, 96822, USA; 2 National Center for Biotechnology Information, NLM, National Institutes of Health, Bethesda, Maryland, 20894, USA; 3 TEDA School of Biological Sciences and Biotechnology, Nankai University, Tianjin, 300457, PR China; 4 Institute of Geological and Nuclear Sciences, Wairakei Research Centre, Taupo, New Zealand; 5 Department of Biological Sciences, University of Calgary, 2500 University Dr. NW, Calgary, Alberta, T2N 1N4, Canada; 6 Key Laboratory of Molecular Microbiology and Technology of the Ministry of Education, College of Life Sciences, Nankai University, Tianjin, 300071, PR China; 7 Department of Microbiology, University of Hawai'i, Snyder Hall, 2538 The Mall, Honolulu, Hawaii, 96822, USA; 8 B-6, Bioscience Division, MS M888, Los Alamos National Laboratory, Los Alamos, NM 87545, USA

## Abstract

**Background:**

The phylum *Verrucomicrobia *is a widespread but poorly characterized bacterial clade. Although cultivation-independent approaches detect representatives of this phylum in a wide range of environments, including soils, seawater, hot springs and human gastrointestinal tract, only few have been isolated in pure culture. We have recently reported cultivation and initial characterization of an extremely acidophilic methanotrophic member of the *Verrucomicrobia*, strain V4, isolated from the Hell's Gate geothermal area in New Zealand. Similar organisms were independently isolated from geothermal systems in Italy and Russia.

**Results:**

We report the complete genome sequence of strain V4, the first one from a representative of the *Verrucomicrobia*. Isolate V4, initially named "*Methylokorus infernorum*" (and recently renamed *Methylacidiphilum infernorum*) is an autotrophic bacterium with a streamlined genome of ~2.3 Mbp that encodes simple signal transduction pathways and has a limited potential for regulation of gene expression. Central metabolism of *M. infernorum *was reconstructed almost completely and revealed highly interconnected pathways of autotrophic central metabolism and modifications of C_1_-utilization pathways compared to other known methylotrophs. The *M. infernorum *genome does not encode tubulin, which was previously discovered in bacteria of the genus *Prosthecobacter*, or close homologs of any other signature eukaryotic proteins. Phylogenetic analysis of ribosomal proteins and RNA polymerase subunits unequivocally supports grouping *Planctomycetes*, *Verrucomicrobia *and *Chlamydiae *into a single clade, the PVC superphylum, despite dramatically different gene content in members of these three groups. Comparative-genomic analysis suggests that evolution of the *M. infernorum *lineage involved extensive horizontal gene exchange with a variety of bacteria. The genome of *M. infernorum *shows apparent adaptations for existence under extremely acidic conditions including a major upward shift in the isoelectric points of proteins.

**Conclusion:**

The results of genome analysis of *M. infernorum *support the monophyly of the PVC superphylum. *M. infernorum *possesses a streamlined genome but seems to have acquired numerous genes including those for enzymes of methylotrophic pathways *via *horizontal gene transfer, in particular, from *Proteobacteria*.

**Reviewers:**

This article was reviewed by John A. Fuerst, Ludmila Chistoserdova, and Radhey S. Gupta.

## Background

The phylum *Verrucomicrobia *is an intriguing but poorly characterized group of bacteria. Representatives of this phylum have been found in a wide range of habitats including soils, aquatic systems, marine sediments, and hot springs; some even occur as endosymbionts [1]. Although various members of *Verrucomicrobia *are estimated to constitute up to 10% of all bacteria in soil, very few have ever been grown in culture [1] and little is understood about their ecological role(s) in the environment. Recent phylogenetic analyses of 16S rRNA sequences suggest that *Verrucomicrobia *form a clade with *Planctomycetes*, *Chlamydiae*, *Lentisphaerae*, *Poribacteria*, and OP3. This putative bacterial clade, which is referred to as the PVC superphylum, unites organisms with a remarkably broad range of lifestyles, from intracellular parasites with some of the smallest known genomes to complex soil organisms [1,2].

We have recently isolated an extremely acidophilic and thermophilic methanotroph belonging to the phylum *Verrucomicrobia*, which was have tentatively named "*Methylokorus infernorum" *strain V4 [3]. This bacterium was isolated from a soil sample in Hell's Gate (Tikitere), a methane-emitting geothermal field in the North Island of New Zealand. The organism grows optimally at pH between 2.0 to 2.5 and temperature of 60°C when supplemented with 25% (v/v) methane as the sole source of energy. Two additional isolates of *Verrucomicrobia*, also with thermoacidophilic phenotypes and greater than 98% 16S rRNA sequence similarity, were concurrently isolated from other geothermal areas: "*Acidimethylosilex fumarolicum" *strain SolV from Solfatara volcano mudpot in Italy, and "*Methyloacida kamchatkensis" *strain Kam1 from an acidic hot spring in Kamchatka, Russia [4,5]. The three methanotrophic *Verrucomicrobia *isolates [3-5] are now being proposed collectively to represent the genus '*Methylacidiphilum*' (manuscript in preparation). Isolate V4 will be proposed under the name '*Methylacidiphilum infernorum*'. Since none of these isolates has been formally described so far, we use designations 'strain V4' and "*M. infernorum*" interchangeably in this paper, although the appropriate name for the organism at this time should be "*Candidatus *Methylacidiphilum infernorum" [6].

Aerobic methanotrophic bacteria thrive in surface sediments of wetlands, lakes and oceans, as well as in sewage sludge and soils. Until recently, all cultivated species belonged to the *Alpha*- and *Gamma*- classes of the phylum *Proteobacteria *(reviewed in [7]). Although some acidophilic proteobacterial methanotrophs have been isolated, none of these grow optimally below pH 5 [8-10]. Together, the three *Verrucomicrobia *isolates form the only known group of aerobic methanotrophs outside of the *Proteobacteria *phylum, and are by far the most acidophilic bacteria capable of methane oxidation [3-5].

Failure of standard primer sets and radioactive probes to detect key enzymes involved in methanotrophy in two of the recent studies of methanotrophic *Verrucomicrobia *[3,5] emphasizes the importance of complete genome sequencing for elucidating the physiology of these previously unknown methane oxidizers. Here, we report the complete genome sequence and annotation of the first methanotrophic bacterium of the *Verrucomicrobia *phylum. In our previous report, based on the draft genome sequence of *M. infernorum *[3], we proposed that certain C_1 _metabolic pathways were common with proteobacterial methanotrophs, while other pathways were incomplete or missing. Here, we report the complete genome sequence, explore evolutionary provenances of *M. infernorum*, present a full reconstruction of the central metabolism of this organism and propose possible pathways involved in methanotrophy.

## Results and Discussion

### Genome organization

**Figure 1:**
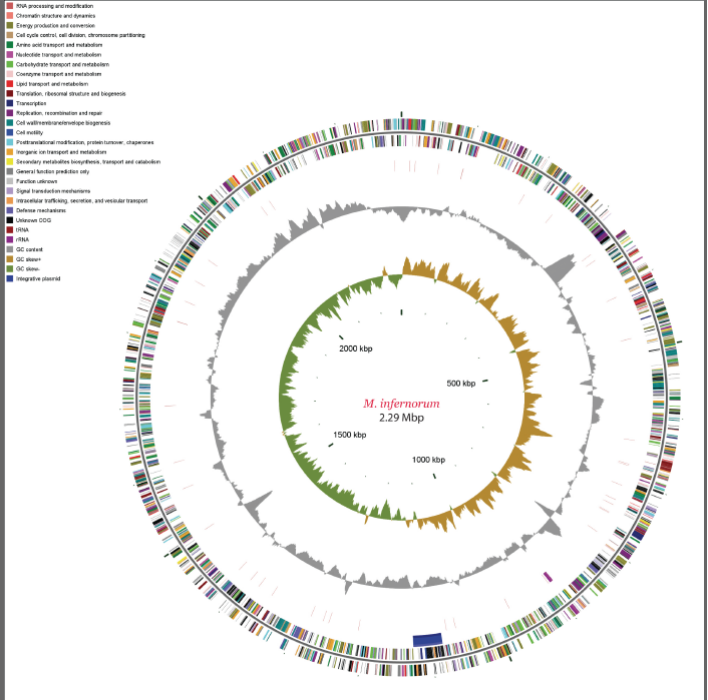
**Circular representation of the "*Methylacidiphilum infernorum*" V4 genome**. The first and second circles show coding regions in positive and negative strands classified by COG functional categories. Potential integrative plasmid region is shown as a blue segment. The third circle shows tRNA and rRNA coding genes. The fourth circle shows variations in G+C content of the genome with respect to the mean G+C value. The fifth circle shows GC-skew plot of the genome showing approximate origin of replication and termination sites.

The genome of *M. infernorum *strain V4 [GenBank: CP000975] consists of a single circular chromosome of 2,287,145 bp. General features of the genome and a summary of the annotation of protein-coding genes are shown in Table 1 and Figure 1. The origin of replication was identified by GC skew analysis [11] and was mapped 250 nt upstream the *dnaA *gene. We identified approximately 20 loci that correspond to a single class of insertion sequences of the IS605 family [12]. No prophages were detected but there is a region in the genome that comprises a potential integrative plasmid (Minf_1153–Minf_1199). Among the 2473 predicted proteins, 731 had no detectable homologs in the NCBI protein database. This fraction of "ORFans" is similar to those reported for the first sequenced genomes from other bacterial phyla and is consistent with the notion that *Verrucomicrobia *form a distinct clade that is only distantly related to other bacteria. Most of the proteins that had homologs in the database could be assigned to the Clusters of Orthologous Groups of proteins (COGs, [13]), see Table 1.

**Table 1:** General properties of "*Methylacidiphilum infernorum*" genome

**Feature**	"*Methylacidiphilum infernorum*" V4
Genome size	2,287,145 bp
G+C content	45.5%
Protein coding genes (CDSs)	2473
Average size of CDSs, bp	841
Percentage coding, %	91.2%
Proteins with known or general biological function	1522 (61%)
Proteins assigned to COGs	1542 (62%)
Hypothetical proteins (no similarity to any proteins)	731
tRNA	46
rRNA (23S, 16S and 5S)	1 operon
Small RNA	3
Riboswitches	2
CRISPR repeats	25 repeats
Transposons	~9
Possible intergrated plasmid	Minf_1152 – Minf_1200

### Phylogeny of "*Methylacidiphilum infernorum*" V4

**Figure 2:**
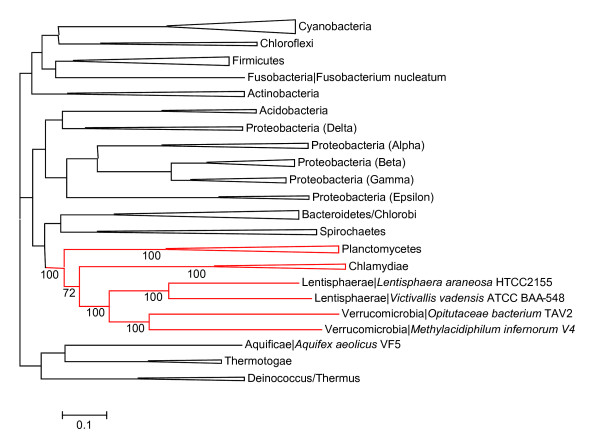
**Maximum Likelihood phylogenetic tree of bacteria constructed from concatenated alignment of ribosomal proteins**. Phylogenetic tree of 59 selected bacterial species (listed in the Supplementary Table 1 [see Additional file 1]) was constructed from concatenated alignments of 51 ribosomal proteins. Bootstrap values are shown only for members of the *Planctomycetes/Verrucomicrobia/Chlamydiae *superphylum.

Analysis of the 16S rRNA sequence of *M. infernorum *identified it as a member of a new subdivision in the phylum *Verrucomicrobia *[3], see also [4,5]. Ever since the original description of the *Verrucomicrobia *as a separate lineage of bacteria [14], analysis of 16S rRNA sequences showed clustering of *Verrucomicrobia *with *Planctomycetes *and/or *Chlamydiae *but the bootstrap support for this grouping was typically less than 50% [15,16]. Similar results were obtained from the analysis of 23S rRNA sequences [17]. Recent phylogenetic analyses of 16S rRNA and ribosomal protein sequences have led to the proposal that four bacterial phyla; namely *Planctomycetes*, *Verrucomicrobia*, *Chlamydiae *and *Lentisphaerae*, together with two candidate phyla; *Poribacteria *and OP3, comprise a single superphylum [1,2]. However, analysis of phylogenetic trees and shared inserts in several conserved proteins has confirmed the affinity of *Chlamydiae *and *Verrucomicrobia *but failed to support the affiliation between these phyla and the *Planctomycetes *[18]. Having determined the complete genome sequence of *M. infernorum *and with complete genomes also available for representatives of *Chlamydiae *and *Planctomycetes*, we constructed phylogenetic trees for concatenated sequences of ribosomal proteins and subunits of the RNA polymerase. In both trees, *M. infernorum *confidently grouped with *Chlamydiae*, and the *Verrucomicrobia-Chlamydiae *clade grouped with *Planctomycetes *(Figure 2 and Supplementary Figure 1 [see Additional file 1]).

**Figure 3:**
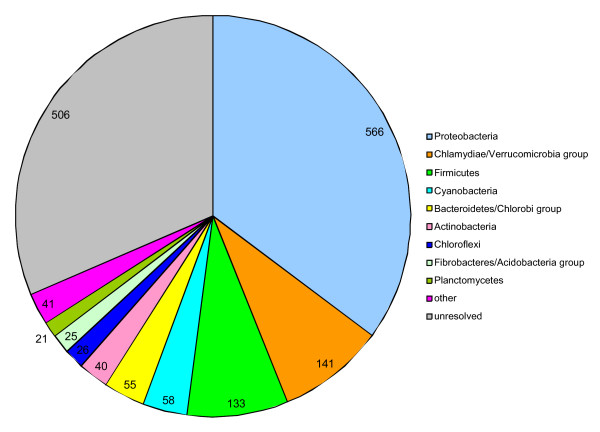
**Taxonomic affiliations of "*Methylacidiphilum infernorum*" V4 proteins**. Taxonomic affiliations of the best BLAST hits for each of the *M. infernorum *proteins to the RefSeq database were analyzed as described in the Methods section.

However, sequence analysis of the complete set of predicted proteins of *M. infernorum *revealed a complex picture. The largest fraction (~23% of the proteins) had their top BLAST hits among proteobacterial proteins, whereas the fraction of proteins that were most similar to homologs from the *Verrucomicrobia*/*Chlamydiae *and *Planctomycetes *group comprised only ~7% (Figure 3). These observations must be interpreted with caution considering the unequal representation of bacterial phyla in current databases (with considerable over-representation of *Proteobacteria*) as well as the fact that sequence similarity is not necessarily an accurate reflection of phylogenetic affinity. Nevertheless, with these caveats, and considering the availability of several large (albeit, with the exception of *Rhodopirellula baltica*, unfinished) planctomycete genomes in the database used for *M. infernorum *genome analysis, the broad spread of the top hits seems to suggest a complex history of this lineage, with numerous putative horizontal gene exchanges shaping the genome. The abundance of proteins with the greatest similarity to homologs from *Proteobacteria *is compatible with the dominance of this bacterial phylum among the known methylotrophs.

**Figure 4:**
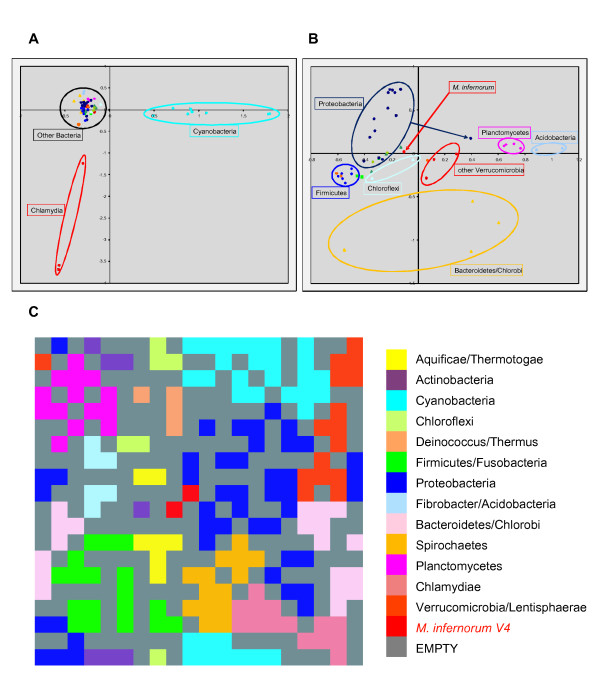
**Analysis of eggNOG phyletic patterns of proteins encoded in various bacterial genomes**. The plot shows the position of individual genomes on the plane of the first two principal components. Major bacterial groups are indicated. A. All 59 bacteria (Supplementary Table 1 [see Additional file 1]). B. Cyanobacteria and Chlamydia removed from the dataset. C. Self-organizing map of the genome content.

To compare the overall gene composition in *M. infernorum *with other bacteria, we performed Correspondence Analysis of the matrix of the phyletic patterns (presence or absence of the given gene in a given genome) of 59 bacterial species (listed in Supplementary Table 1 [see Additional file 1]) in the eggNOG database [19]. The results show that *M. infernorum *groups neither with its closest relatives (*Chlamydiae *and *Verrucomicrobia*) nor with any other bacterial clade (Figure 4). This lack of clustering by phyletic pattern together with the position of *M. infernorum *(Figure 4B) near the origin of coordinates (which, by definition, is the baricenter of the data set) further supports the notion of a complex history of the gene set of *M. infernorum*, with likely contributions from diverse groups of bacteria. The nearest neighbors of *M. infernorum *in the genome content space (Figure 4C) are various members of *Proteobacteria *(*Rickettsia*, *Neisseria*, *Escherichia*, *Methylococcus*), *Thermotogae*, *Aquificae *and a single representative of *Actinobacteria *(*Rubrobacter*).

Our attempts to identify a genomic signature for the *Planctomycetes/Verrucomicrobia*/*Chlamydiae *superphylum, i.e., a set of genes that would be present in all members of this group but in no other organisms, failed to identify any genes fitting that definition. The closest candidate was a protein of unknown function, Minf_1886, which is present in *Verrucomicrobia *and *Planctomycetes *but not in *Chlamydiae *(Supplementary Figure 2 [see Additional file 1]). Similarly, of the 12 protein families that recently have been reported to be specific for the PVC superphylum [2], only four were identified in *M. infernorum *(Supplementary Table 2 [see Additional file 1]), and representatives of all of these families could also be found in other bacterial clades (data not shown).

### Evolution of the "*Methylacidiphilum infernorum*" V4 branch

Since the gene content of *M. infernorum *substantially differs from the gene contents of other member of the PVC superphylum, we used the inferred gene set of the last common ancestor of all bacteria (LCBA) to reconstruct the most parsimonious scenario of gene gain and loss in this branch [20]. This approach assigned 1382 COGs (genes) to the LCBA [21]. The results of the reconstruction for the *M. infernorum *lineage suggest considerable gene flux dominated by gene loss (526 genes inferred to have been lost and 262 gained). Approximately 75 genes might have been lost at the level of the last common ancestor of the PVC superphylum, including cell division proteins FtsX, FtsE, MinC, MinD, and MinE. Predicted gene gains additionally encompassed *M. infernorum *proteins that did not fit into any COGs including most of the genes responsible for methylotrophy. There are approximately 200 such proteins that have homologs in the databases and 731 ORFans. In accord with the lifestyle of *M. infernorum*, it appears that many genes involved in autotrophy were gained whereas genes related to heterotrophic processes were lost (Supplementary Figure 3 [see Additional file 1]). This dynamic was especially prominent among the genes coding for proteins implicated in energy metabolism, where approximately equal numbers of genes have been lost and gained. In most other metabolism-related categories, gene loss exceeded gene gain.

Notably, many regulatory and even informational genes were apparently lost, a trend that might reflect ongoing genome streamlining, especially considering that *M. infernorum *has the smallest genome among all representatives of *Verrucomicrobia *with known or estimated genome sizes. Some of the apparent gains and losses appear quite unexpected. In particular, *M. infernorum *seems to have acquired genes for several proteins that belong to the gene set that is conserved in archaea and eukaryotes. This group of proteins includes three subunits of the proteasome (Minf_1279, Minf_1281 and Minf_1284), and accessory and regulatory proteins encoded in the same neighborhood, ATP-dependent DNA ligase (Minf_0008, COG1423), and archease, a protein apparently involved in diverse nucleic acid modification reactions (Minf_0305, COG1371). These proteins of *M. infernorum *are most closely related to orthologs encoded in other bacteria; in particular, the proteasome subunits show the strongest similarity to orthologs from *Actinobacteria*. These observations suggest extensive horizontal gene transfer among bacteria, conceivably, following the initial transfer from an archaeal source.

The specific gene loss in the *M. infernorum *branch appears to be another manifestation of genome streamlining. Several highly conserved informational and housekeeping genes are encoded in all other sequenced genomes of the PVC superphylum, but not in *M. infernorum*. This group of lost genes includes those encoding the house-cleaning protein Maf (COG0424), rRNA methylase SpoU (COG0566); tRNA and rRNA cytosine-C^5^-methylase Sun (COG0144), tRNA-dihydrouridine synthase (COG0042), single-stranded DNA-specific exonuclease RecJ (COG0608), and the type II secretory system PulDFG. Several genes, in particular, those for proteins involved in DNA repair, apparently have been lost independently in both the *M. infernorum *branch and the *Chlamydiae *branch (these genes are present in other genomes from the *Verrucomicrobia/Lentisphaerae *branch). These include SbcC (COG0419) and SbcD (COG0420), respectively, an ATPase and exonuclease involved in repair of stalled replication forks, and RadC (COG2003), implicated in recombinational repair. The absence of these proteins in *M. infernorum *is unexpected because they are encoded in the genomes of the great majority of free-living bacteria.

Despite the general trend toward gene loss, we identified several lineage-specific expansions of paralogous gene families in the *M. infernorum *genome. Several of these expanded families encode membrane proteins. There are at least 19 paralogs of a TonB-like outer membrane receptor (COG1629) that is involved in import of essential organometallic micronutrients, including iron-siderophores [22]. There are also 10 clusters coding for the outer membrane channel protein TolC (COG1538) and/or the periplasmic (fusion) protein AcrA (COG0845), which might be involved in multidrug or heavy-metal efflux [23]. Another expansion includes 6 paralogs of the starvation-inducible outer membrane lipoprotein Slp (COG3065) [24]. Expansion of these protein families is typical of proteobacterial methylotrophs and *Proteobacteria *in general; however, expansion of the Slp family might be related to acid resistance of *M. infernorum *(see below).

### Reconstruction of *M. infernorum *V4 metabolism and adaptations to methylotrophy

**Figure 5:**
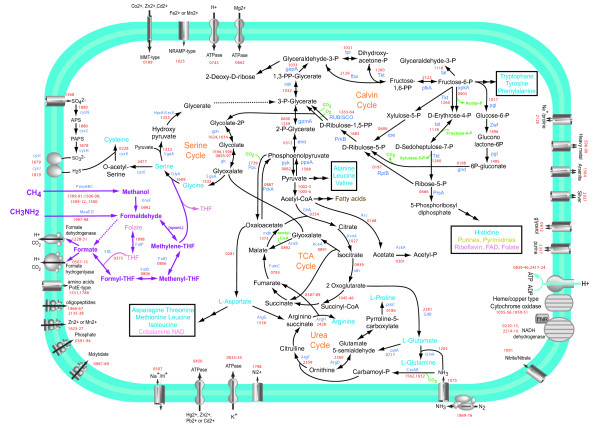
**Reconstruction of methanotrophic and central metabolism pathways of "*Methylacidiphilum infernorum*" V4**. For each predicted reaction, the generic gene name (blue) and *M. infernorum *V4 gene identifier (red, "Minf" prefix is omitted) are shown. The methylotrophy-related pathways are shown by magenta. Accessory products and metabolites are shown be green. Reactions for which no candidate enzyme was confidently predicted are indicated by dashed arrows. Key metabolites are shown as follows: light blue for amino acids, dark yellow for nucleotides, brown for sugars, pink for cofactors. Abbreviations: THF – tetrahydrofolate, CoA – coenzyme A.

Analysis of the *M. infernorum *genome allowed us to reconstruct its central metabolic pathways and mechanisms of methane utilization (Figure 5).

#### Central metabolic pathways and their variations

At less than 2.3 Mbp, the *M. infernorum *genome is much smaller than the genomes of proteobacterial methylotrophs *Methylobacillus flagellatus*, *Methylobacterium extorquens*, *Methylococcus capsulatus*, and *Methylibium petroleiphilum *[25-28]. Accordingly, *M. infernorum *appears to encode only a core set of enzymes required for methylotrophic growth but lacks genes for enzymes of carbohydrate utilization that are present in some of those organisms. Despite its small (for a non-parasitic bacterium) genome size, *M. infernorum *is predicted to possess most of the key metabolic pathways for the biosynthesis of all amino acids, nucleotides and cofactors, with the sole exception of the cobalamin cofactor (Supplementary Table 3 [see Additional file 1]). Several key enzymes of these pathways were represented by unusual enzyme forms, for example, in the biosynthesis of folate, the cofactor that is necessary for C_1 _transfer reactions. Although the classical GTP cyclohydrolase FolE, which is responsible for first step in the folate biosynthesis pathway, appears to be missing, *M. infernorum *encodes an alternative GTP cyclohydrolase (Minf_0065), a recently characterized enzyme that belongs to COG1469 [29]. Likewise, dihydrofolate reductase FolA that catalyzes the last step of the pathway appears to be missing but its function is likely taken over by dihydropteroate synthase FolP (Minf_1898), as shown recently for *Helicobacter pylori *[30].

In contrast to all other methylotrophs, *M. infernorum *employs the 3-dehydroquinate dehydratase of COG0710 (AroD type) but not COG0757 (AroQ type) in the biosynthesis of aromatic amino acids. Similarly, for lipoic acid biosynthesis, it employs lipoate-protein ligase A but not lipoate-protein ligase B as seen in other methylotrophs. Asparagine synthases of both classes (COG0367 and COG2502) are missing in *M. infernorum*, so asparagine is likely to be formed by transamination [31]. Threonine dehydratase that is responsible for the first step of isoleucine biosynthesis is missing but the product of this reaction, α-ketobutyrate, can be produced from pyruvate and acetyl-CoA via a three-step pathway involving *leuBCD *gene products. *Methylacidiphilum infernorum *V4 can fix ammonia both through the glutamine synthesis reaction and through the carbomoyl-phosphate synthesis reaction. The latter substrate is used in the urea cycle, for which all genes are present except for the gene for arginase, which cleaves arginine into urea and ornithine. However, *M. infernorum *encodes 4-aminobutyrate aminotransferase ArgD that can ultimately supply ornithine back to the cycle through a part of the TCA cycle and glutamate synthesis (Figure 5). Other methylotrophs possess neither arginase nor ArgD. In addition to assimilating ammonia, *M. infernorum *should be able to fix gaseous nitrogen, as it possesses a complete set of genes for nitrogen fixation. The genome encompasses a gene cluster for iron-molybdenum-dependent nitrogenase (Minf_1869–76) and two additional clusters that contain genes for biogenesis of cofactors, scaffolding and electron transfer proteins (Minf_0453–463, Minf_0465–477), as well as a Mo/Fe-nitrogenase-specific transcriptional regulator NifA (Minf_0464). Most of these genes and their organization in putative operons are very similar to those of *Methylococcus capsulatus *[28], a methanotroph that has been shown to fix nitrogen (see [32] and references therein).

Another notable difference between *M. infernorum *and other methylotrophs is the number and diversity of transporters encoded in their genomes. Even when the genomic data are normalized for its smaller genome size, *M. infernorum *encodes fewer transporters than any of the other four completely sequenced genomes of methylotrophs, as well as many other bacteria (Supplementary Figure 4 [see Additional file 1]). A similar pattern is seen with transcriptional regulators, which apparently have been lost during the evolution of *M. infernorum *branch (Supplementary Figures 3 and 5 [see Additional file 1]).

#### Pathways involved in methanotrophy

We identified three *pmoCAB *operons that encode the three subunits of particulate membrane-bound methane monooxygenase (pMMO). Two of these operons are adjacent to one another (Minf_1506–Minf_1511) in the *M. infernorum *genome. As in *Methylococcus capsulatus*, a separate (fourth) copy of pMMO subunit C is encoded in a different locus (Minf_1500), suggesting a somewhat different role for this particular subunit. We have shown previously that the three β (PmoA) subunits of *M. infernorum *pMMO form a distinct branch in the corresponding phylogenetic tree and probably evolved via lineage-specific duplications [3]. No genes for soluble form of methane monooxygenase (sMMO) were found.

Methanol is a product of methane oxidation and also can be available from the exogenous sources [7]. A homologue of *mxaF *(or *xoxF*), encoding the methanol dehydrogenase large subunit (Minf_0992), was identified in the *M. infernorum *genome together with genes for two proteins required for its catalytic function: a methanol-binding periplasmic protein (Minf_0995) and a cytochrome c family protein (Minf_0996). However, neither the small subunit of methanol dehydrogenase gene *mxaI *nor genes for several accessory proteins found in *M. capsulatus *and other methylotrophs were detected [3]. The lack of *mxaI *is not surprising because the genomes of several other methylotrophic bacteria including *Rhodobacter sphaeroides*, *Methylibium petroleiphilum*, and the β-proteobacterium HTCC2181 lack this gene as well [27,33,34]. *Methylibium petroleiphilum *and HTCC2181 possess other *mxa *accessory genes that were not found in the genome of *M. infernorum *but *Rhodobacter *oxidizes methanol while possessing only a similar gene complement to *M. infernorum*. The genes for enzymes of biosynthesis of PQQ, the methanol dehydrogenase cofactor, are all present (PqqABCDE cluster, Minf_1233–1237). In line with the trend of genome streamlining, *M. infernorum *has only a single gene (Minf_1885) for PQQ biosynthesis peptidase (PqqL/PqqF/PqqG family), as opposed to two peptidase genes in the genomes of other methylotrophs [25-27].

In other methanotrophs, two pathways of fixation of formaldehyde, a product of the reaction catalyzed by methanol dehydrogenase, have been characterized [7]. Hexulose-6-phosphate synthase and hexulose-phosphate isomerase, key enzymes of the ribulose monophosphate (RuMP) pathway, were not detected in *M. infernorum*. In addition, two distal enzymes of the assimilatory branch of the RuMP pathway (6-phosphogluconate dehydratase, Kdd, and phospho-2-keto-3-deoxygluconate aldolase, Eda) are also missing in *M. infernorum*. This observation shows that *M. infernorum *does not use the RuMP pathway for formaldehyde assimilation, which has been reported previously for several other methanotrophs, such as *Methylosinus *and *Methylocystis *[7].

Another route of formaldehyde fixation commonly used by methylotrophic bacteria is the serine pathway. This pathway involves pyridoxal phosphate- and tetrahydrofolate-dependent serine hydroxymethyltransferase, which produces serine from formaldehyde and glycine. Subsequently, serine can be metabolized into 3-phosphoglycerate and further used for biomass production. We identified serine hydroxymethyltransferase GlyA and most of the other enzymes of serine pathway described for the methylotroph *Methylobacterium extorquens *[25], except for malyl coenzyme A lyase (COG2301) and glycerate kinase (COG1929 or COG2379 or COG4240). Malyl coenzyme A lyase cleaves malyl-CoA, yielding glyoxylate, which in turn can be converted to glycine, so serine cycle can start again. Glycerate kinase (COG1929 or COG2379 or COG4240) converts glycerate to 3-phosphoglycerate, another essential reaction of the serine cycle. The absence of these key enzymes suggests that alternative routes for the completion of the serine cycle exist in *M. infernorum*.

Our reconstruction predicts at least two possible routes to form glyoxylate. One is *via *the Calvin cycle, i.e., the oxygenation reaction of ribulose-bisphosphate carboxylase [35], yielding phosphoglycolate, which is converted into glyoxylate. Another route is *via *the downstream reactions of glycolysis followed by the glyoxylate shunt. This pathway is absent in other methanotrophs but might be the main route leading to glyoxylate regeneration in *M. infernorum *(Figure 5), as proposed earlier [3]. We did not detect genes specific for the glyoxylate regeneration cycle and the associated poly-β-hydroxybutyrate (PHB) pathway that has been characterized in some other methylotrophs [25]. However, *M. infernorum *encodes an unusual protein (Minf_1669) that contains a serine hydroxymethyltransferase domain and two additional domains that are homologous, respectively, to low molecular weight phosphatase and ribose-5-phosphate isomerase B. The domain composition of this protein suggests a tight connection between the serine pathway and the pentose phosphate pathway. In addition to methane oxidation, *M. infernorum *possesses some of the genes, albeit not all, that are required to utilize methylamine *via *the methylamine dehydrogenase system [36,37]. To date, however, we have not been successful in growing the culture on methylamine as the sole substrate [3].

Formaldehyde oxidation pathways are present in all known organisms capable of growth on methane and methanol and appear to be essential for energy generation during methylotrophic growth [25,38]. All these organisms have pathways for transferring C_1 _units between the oxidation levels of formaldehyde and formate. All the pathways require folate cofactors and include one or more formate dehydrogenase complexes. Although *M. infernorum *encodes the complete formaldehyde oxidation pathway, it does not have the methylene-H4F dehydrogenase and methenyl-H4F cyclohydrolase enzymes that have been characterized in other methylotrophs. Instead, like many other bacteria, it apparently uses the *folD *gene product to perform the same reactions (Figure 5). The tetrahydromethanopterin cofactor-based "archaeal" pathway for C1 transfer found in all other methylotrophs [25-27,39] is not present in the *M. infernorum *genome.

### Degradation of the cell division machinery

Like other members of the PVC superphylum, *M. infernorum *shows gene loss and alteration of multiple components of the cell-division protein machinery. As indicated above, genes for FtsX, FtsE, MinC, MinD, and MinE have been lost in the *M. infernorum *lineage, whereas the *ftsZ *gene apparently has experienced a period of accelerated evolution (data not shown) as previously demonstrated for the *ftsZ *genes of several *Prosthecobacter *species, which also belong to the *Verrucomicrobia *[40]. In contrast, the rate of evolution of *ftsK *and *ftsA *genes did not seem to be affected (data not shown). Unlike *Prosthecobacter dejongeii *[41,42], *M. infernorum *does not encode tubulin or homologs of any other distinctive eukaryotic proteins involved in cell division or cytoskeletal functions. Both *Chlamydia *and *Planctomycetes *show morphological correlates of the altered cell-division apparatus, a unique condensed form of chromatin in the former and a striking, nucleus-like enclosure containing the chromosome in the latter [43,44]. The observations on the unusual planctomycete cell morphology, some hints from planctomycete genome analysis, and the finding of tubulin in *Prosthecobacter dejongeii *have led to speculation on the potential relevance of the cell division mechanism in this group of bacteria to evolution of the eukaryotic nucleus and cytoskeleton [45]. Neither detailed analyses of the planctomycete genomes.([46,47] nor the present analysis of the first completed verrucomicrobial genome provide any support for this idea. However, it does seem that there was a major alteration of cell division mechanisms at the onset of evolution of the PVC superphylum, with subsequent elaborations in individual lineages [48], and experimental study of division in these bacteria will be of major interest.

### Adaptation of *M. infernorum *to the acidic environment

*M. infernorum *encodes a glutamate decarboxylase (Minf_0102) and a potential glutamate/γ-aminobutyrate antiporter (Minf_1788), as well as an arginine decarboxylase (biosynthetic, Minf_2107) and a potential arginine/agmatine antiporter (Minf_1531). These enzyme/transporter pairs could potentially counteract acidification of the cytoplasm by binding excess H^+ ^ions and releasing CO_2 _[49]. Another potential acid-resistance mechanism that could be utilized by *M. infernorum *is agmatine hydrolysis by agmatine deiminase (Minf_0964), which releases NH_3 _that would bind excess H^+ ^ions [50]. *M. infernorum *encodes neither urease, which accounts for acid resistance in *Helicobacter pylori *[51], nor orthologs of *E. coli *proteins HdeA, HdeD, YhiD, and YhiF that are also implicated in acid resistance. However, *M. infernorum *carries two paralogs of the Na^+^/H^+ ^antiporter NapA [52] and six paralogs of the gene coding for the starvation-inducible outer membrane lipoprotein Slp [24], which has an unknown function but is often co-expressed with acid-resistance genes [49].

The *M. infernorum *genome also carries two operons encoding H^+^-translocating F_1_F_O_-ATPase subunits, the first such case among all sequenced microbial genomes. One of these operons (Minf_2417–Minf_2424) is most similar to the ATPase operons of other members of *Verrucomicrobia *(data not shown) and probably represents the form of the enzyme that is ancestral to the PVC superphylum. By contrast, the second ATPase operon (Minf_0839–Minf_0846) is most similar to the ATPase genes from gamma-proteobacteria, such as *Methylococcus capsulatus *(Supplementary Figure 6 [see Additional file 1]), and might have been acquired by a relatively recent lateral gene transfer. These enzymes are reversible, so it remains to be determined whether they synthesize ATP by utilizing proton gradient (allowing influx of protons into the cell) or couple ATP hydrolysis with pumping protons out of the cell.

**Figure 6:**
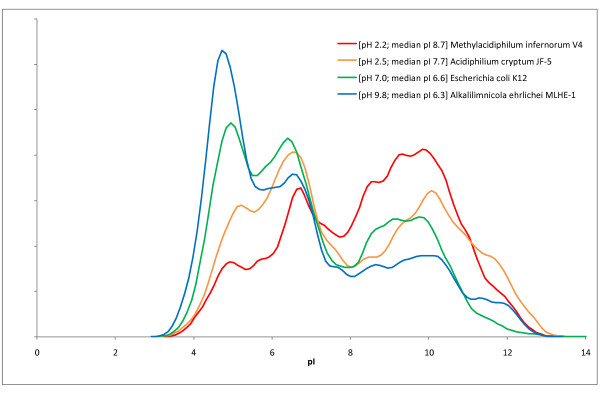
**Adaptation of "*Methylacidiphilum infernorum*" V4 to the acidic environment**. Distribution (estimated probability density function) of isoelectric points of proteins encoded in genomes of microorganisms with different pH preferences.

Adaptations to the extremely acidic environment are also seen in the amino acid composition of *M. infernorum *proteins. The distribution of isoelectric points of *M. infernorum *proteins shows a substantial excess of high-pI (basic) proteins (Figure 6) and is much more similar to that of other acidophiles (e.g. *Acidiphilium cryptum *JF-5) than to that of mesophiles (*Escherichia coli *K12) or alkaliphiles (*Alkalilimnicola ehrlichei *MLHE-1).

Like some other extreme acidophiles, *M. infernorum *encodes a relatively simple signal transduction system that includes 8 sensor histidine kinases and 10 response regulators (with 7 pairs clustered in operons), but no Ser/Thr protein kinases, adenylate or diguanylate cyclases, or chemotaxis receptors. Although *M. infernorum *might be capable of gliding motility (using Minf_0409–Minf_0411 proteins), it has no chemotaxis genes.

### Antiviral and stress-response systems

Similarly to most other thermophiles, *M. infernorum *has the CRISPR-associated system, which is involved in anti-phage defense [53,54]. The system contains a predicted polymerase cassette (the polymerase itself is encoded by Minf_0882 gene) that seems to be a thermophilic determinant, i.e. is found primarily in thermophiles [53,55]. Despite the presence of 6 clusters of CRISPRs (altogether 25 repeats), the system might be inactive considering that that *cas1 *gene (Minf_0870) that is ubiquitous in CRISPR-associated systems is truncated. Another system often found in thermophiles is a pair of proteins containing a minimal nucleotidyltransferase and the HEPN (COG2250) domain that might be a novel toxin-antitoxin system (KSM, YIW, EVK, unpublished observations). The latter proteins also have a role in phage defense and stress response [56], along with the better studied systems of restriction-modification [57]. We identified a few toxin-antitoxin components in *M. infernorum *(Minf_0121, Minf_0349, Minf_1374) and a classic restriction-modification system (Minf_1805 and Minf_1806). There is an additional locus encoding DNA methylases, helicases and a nuclease that might have a similar role (Minf_0316–328).

*M. infernorum *possesses an elaborate system of heavy-metal resistance. Along with the expansion of heavy metal efflux systems mentioned above, we identified a mercury reduction system (Minf_0449–Minf_0451), arsenate reductase (Minf_1582), putative silver efflux pump (Minf_2037), and a tellurium resistance protein (Minf_2102).

## Conclusion

*"Methylacidiphilum infernorum" *V4 has a streamlined genome with a gene complement typical of an autotrophic bacterium, simple signal transduction pathways and limited potential for regulation of gene expression. With only a few gaps, reconstruction of the central metabolism was complete and straightforward including pathways involved in methanotrophy. Phylogenetic analysis of informational molecules unequivocally supports grouping the *Chlamydiae*, *Planctomycetes*, and *Verrucomicrobia *into a superphylum. An important feature of this clade is the alteration and partial degradation of the cell division apparatus that is manifest in the loss of several genes and acceleration of evolution of others. However, analysis of the entire complement of *M. infernorum *genes seems to tell a different story, namely, one of extensive horizontal gene exchange with a variety of bacteria. The genome of *M. infernorum *shows many apparent adaptations for existence under extremely acidic conditions including a major shift in the isoelectric points of cytoplasmic proteins.

## Methods

The genome of *M. infernorum *was sequenced using the whole-genome-shotgun approach as previously described [58]. Genomic DNA of *M. infernorum *was randomly sheared to generate 3 kb insert fragments. These fragments were size-selected on agarose gels, purified, end-repaired and ligated to pUC118 vectors and then transformed into DH10B competent cells. Plasmids from positive clones were isolated using Eppendorf Perfect Prep Plasmid 96 Kit (Eppendorf) and sequenced using Beckman CEQ 8000 (Beckman Coulter) and ABI 3730xl (Applied Biosystems) sequencers. A total of 43,008 valid sequences were used for assembly with ARACHNE [59,60], and SEQMAN II (DNAStar) assemblers. Further 344 sequences were used to close gaps between contigs and to improve overall sequence quality of contigs.

ORFs were predicted using GLIMMER [61] followed by BLASTX [62] searches of intergenic regions between predicted ORFs. Transfer RNAs were predicted by tRNAscan-SE [63] and non-coding RNAs were predicted using Infernal software against Rfam database [64]. Genome annotation was performed by running BLAST and PSI-BLAST against the NCBI protein database, COG database [13], and the eggNOG database [19] with manual verification as described previously [58]. Metabolic pathways were analyzed by comparing COG assignments of *M. infernorum *proteins with the standard sets of COGs involved in each pathway [65], using the data from the KEGG database [66].

To analyze the taxonomic affiliations of *M. infernorum *proteins, 10 best BLAST hits (e-value threshold of 0.01) to the RefSeq database (release 29) were collected for each of the *M. infernorum *proteins. Hit score and taxonomic affiliation of the hit at the phylum level were recorded. For each *M*. *infernorum *query, the hits were assigned weights relative to the weight of the best hit (w_i _= (s_i_/s_1_)^32 ^where s_i _is the score of the i-th hit); the weights were summed across the taxa. If the weight for the highest-weight taxon comprised a certain fraction of the total sum of weights (>75%), the query was considered affiliated with this taxon, otherwise the taxon affiliation of the query was considered unresolved. In practical terms, if the second-best hit belonged to a different taxon than the best hit, but its weight was within 3% of that of the best hit, the affiliation of the query would not be considered sufficiently resolved.

Maximum Likelihood trees for the concatenated alignments (positions with >33% of gaps removed) of ribosomal proteins (6137 sites) and RNA polymerase subunits RpoA, RpoB, and RpoC (2625 sites) were constructed using TreeFinder [67], with the Whelan and Goldman [68] evolutionary model and gamma-distributed site rates. Protein isoelectric points were computed using the amino acid pK values from EMBOSS iep program [69].

Analysis of *M. infernorum *genome composition was performed as follows: orthologous sequences from the eggNOG database [19] were aligned and converted to PSI-BLAST [62] PSSMs. These PSSMs were used in PSI-BLAST searches against the genomic databases of 59 bacteria representing all major lineages with completely sequenced genomes (the complete list is given in the Supplementary Table 1 [see Additional file 1], all genome sequences were taken from NCBI's Entrez Genomes database); proteins were assigned to the NOG with the lowest reported e-value [70]. The eggNOG presence/absence table (phyletic patterns) was derived from this data; patterns with less than two species present were removed. The first round of a Correspondence Analysis was performed using the ADE-4 package [71] on the data table comprised of all 59 species (Figure 4A; data projected to the plain of the first two components). The results show that the main trend in the structure of the phyletic pattern space is dominated by the two groups of organisms – *Cyanobacteria *and *Chlamydiae *– that are most different from the rest of the representative genomes. To resolve the structure for the rest of bacteria, the data for cyanobacteria and chlamydia were removed from the table and Correspondence Analysis was performed for the remaining species (Figure 4B). Alternatively, the first 40 principal dimensions of the Correspondence Analysis space were visualized using the Self-Organizing Maps approach [72] on a 20 × 20 lattice with toroidal topology. Euclidean distances between a lattice vector and a data vector were linearly normalized to the range [0..1]. Map nodes were colored according to the taxonomic affiliation of the closest (after normalization) genome vector (Figure 4C).

## Competing interests

The authors declare that they have no competing interests.

## Authors' contributions

MBS, BWM, PFD, EVK, LW and MA designed the study, MBS, BWM, MC, AVS and PFD cultivated the organism and isolated genomic DNA; SH, JHWS, PS, BVL, ZZ, YR, JW, LF and LW performed genome sequencing, KSM, JHWS, MYG, MVO, EVK, YIW and MA performed genome analysis, KSM, MVO, YIW and NY performed phylogenetic analyses, SH, KSM, JHWS, MYG, MVO, YIW, EVK, PFD, LF and MA wrote the paper. All authors read and approved the final manuscript.

## Reviewers' reports

### Reviewer's report 1

John A. Fuerst, School of Molecular and Microbial Sciences, The University of Queensland, Brisbane, Queensland 4072, Australia (nominated by Mark Ragan, The University of Queensland, Brisbane, Australia)

This study reports and analyses the complete genome sequence of an extremely acidophilic methanotrophic member of the distinctive Bacterial phylum *Verrucomicrobia*, "*Candidatus *Methylacidiphilum infernorum" isolate V4, from a New Zealand methane-emitting geothermally heated soil and growing optimally at pH 2.0–2.5 and at 60°C with 25%(v/v) methane as sole source of energy. Related thermoacidophilic organisms have also been isolated recently from other geothermal areas, and these organisms seem to represent a clade of methane-oxidizers in this phylum, a result of significance concerning our understanding of C_1_-compound metabolism and evolution of C_1 _transfer enzymes, since the only known cultivated methane utilizers are members of the phylum *Proteobacteria *and the only phylum other than the proteobacteria in which some C_1_-transfer enzymes (though not functional C_1 _metabolism) have been found is the phylum *Planctomycetes*. Significantly, phylum *Planctomycetes *has been proposed on the grounds of 16S rRNA, 23S RNA and ribosomal protein sequence analysis to be related to the phylum *Verrucomicrobia *members in a single 'PVC" superphylum also proposed to contain phyla *Chlamydiae*, *Lentisphaerae *and the candidate phyla *Poribacteria *and OP3, but the support for the group has been relatively weak, with stronger support for a link between *Verrucomicrobia *and *Chlamydiae *than for either of these phyla with *Planctomycetes*.

Possibly the most significant result of this paper is that the PVC superphylum is supported when concatenated sequences of ribosomal proteins and RNA polymerase subunits are analysed, including "*Candidatus *Methylacidiphilum infernorum". This establishes firmer grounds for more detailed investigation of the relationships and links between members of the constituent phyla, at least for *Planctomycetes*, *Verrucomicrobia *and *Chlamydiae*. It also supports the view that more comprehensive taxon sampling may assist the testing of postulated superphyla, and suggests that genomes of more genera and species representative of all sub-divisions of phylum *Verrucomicrobia *should be sequenced to make this possible and confirm the present analysis. Of course, the recently explored limitations of some concatenated datasets for phylogenetic analysis of deep prokaryote nodes [73] need to be kept in mind. Although this paper reports the first complete genome sequence for a representative of the *Verrucomicrobia*, this thermoacidophilic species might not be representative of other soil, aquatic and symbiotic verrucomicrobia living in less extreme habitats, and this makes achieving completion of the genome sequencing and analysis of members of other subdivisions an urgent priority, including at the least *Verrucomicrobium spinosum*, *Prosthecobacter dejongeii, Chthoniobacter flavus*, *Opitutus terrae*, *Akkermansia muciniphila*, the soil isolate "Ellin514", and the marine verrucomicrobial strain DG1235.

This conclusion concerning a possible superphylum based on analysis of a limited set of informational – i.e. translational and transcriptional – genes is complicated and potentially contradicted by BLAST analysis of the complete set of predicted proteins, which indicated the fraction of proteins most similar to homologs from available genes of the PVC group to be only ~7%, while the largest fraction (~23% of the V4 proteins) had top hits among the phylum *Proteobacteria*. Although the authors admit to certain qualifications concerning over-representation of databases by the *Proteobacteria *and possible poor correlation of sequence similarity with phylogenetic affinity, they nevertheless favour a perspective or inference where horizontal gene transfer (HGT) dominates the architecture of the genome, going even further to link this to proteobacterial methylotrophy (despite the previously published result [3] concerning *pmo *genes for particulate methane monooxygenase needed for methane oxidation indicating that *Methylacidiphilum *genes are completely divergent from those of methylotrophic proteobacteria). The acceptance of such a conclusion may be dependent on the validity of such BLAST analysis, the problems with which in relation to gene transfer detection have been subject to detailed analysis [74-76] and such problems may also apply to this case. There appear to be no other criteria than bioinformatic BLAST or COG analysis to strengthen the conclusion of HGT in this case, and there is then a temptation to consistency with the 'global HGT' dogma without sufficient grounds for high probability that HGT is the only explanation for such results. In other words, there may be other explanations for this apparently contradictory relationship to proteobacteria which need to be considered.

Does phylogenetic analysis of individual genes hypothesized to have been transferred indicate a particular group of proteobacteria from which the transfer may have occurred recently, or is this proposed to be an ancient transfer, in which case how is it to be distinguished from transfers in the progenote? Is the polarity of transfer direction unambiguous? Of course even following such analysis, alternative explanations for phylogenetic misplacement of taxa within an alien clade may also then have to be considered.

To strengthen their conclusions, the authors do include a Correspondence Analysis, a type of gene content analysis, which isolates *M. infernorum *away from either members of phyla *Verrucomicrobia *and *Chlamydiae *or any other bacterial clade. They interpret this as supporting a complex history for the gene set of *M. infernorum*, reflecting contributions of genes from diverse groups, since nearest neighbours in genome content space are members of phylum *Proteobacteria*, *Thermotogae*, *Aquificae *and one *Actinobacteria *genus. Is there an explanation for this analysis alternative to gene transfer? The inclusion of the deep-branching *Thermotogae *and *Aquificae *is interesting, and suggests that one alternative which might be considered is that *Verrucomicrobia*, or at least this representative of the phylum, might harbour gene contributions which either occurred during the early radiation of the Bacteria or even earlier when phyla of the domain Bacteria were not distinguishable. More detailed phylogenetic analysis of the genes used in the genome content analysis may be needed to test such an alternative.

The authors do note the caveats which have to be applied to at least their BLAST analysis, but various methods for confirming the hypothesis of lateral gene transfer such as GC composition, codon usage, or association with possible mechanisms for transfer [76-78] have not yet apparently been applied to the *Methylacidiphilum *genome problem, and these might potentially reinforce the gene transfer explanation derived so far from BLAST and Correspondence Analysis, though it is also possible that ancient transfer would not be detected by such methods.

The situation of *Methylacidiphilum infernorum *bears some similarity to that noted for *Cenarchaeum symbiosum *by Forterre in his review of the analysis of archaeal COGs by Makarova et al. [70] where a gene content analysis indicated possible acquisition of euryarchaeal genes via LGT, in an organism already postulated to have acquired 'lots of bacterial genes' [70]. As in the *C. symbiosum *case where the possibility remains that it represents an early branching archaeal lineage containing bacterial and archaeal homologs lost in other archaea, the alternative hypothesis should be considered that *Methylacidiphilum *and perhaps also other *Verrucomicrobia *(and perhaps even also other members of the PVC superphylum) represent members of an early branching lineage containing ancient homologs of genes in other Bacterial phyla which have been subject to wide (and perhaps even unparsimonious!) loss.

Further tests to estimate the relative timing of the proposed HGT [79] might lead to insights about this possibility. Evidence regarding potential gene loss is presented supporting the interpretation that *M. infernorum *may have acquired genes for several proteins belonging to the gene set conserved in archaea and eukaryotes, but again one asks whether this may alternatively be interpreted not as suggesting HGT among bacteria following initial transfer from archaea but rather as an indication of retention of an ancient signal from an organism close to the LUCA or LCA. Perhaps such an ancient signal could even stem from a lineage analogous to the uncharacterized archaeal lineage recently proposed as a root from which eucaryal archaea-like genes may have originated [80].

The distribution of C_1 _transfer enzymes is intriguing, since the tetrahydromethanopterin-dependent enzymes found in some members of phylum *Planctomycetes *do not seem to have been detected, and *Methylacidiphilum *is clearly capable of methanotrophy unlike any planctomycete so far isolated. The existing controversy [81,82] over the origin of the archaea-like C_1_-transfer enzymes of planctomycetes relating to potential gene transfer versus ancient divergence suggests however that it may be productive to examine the phylogenetic relationships of the C_1_-transfer enzymes of *Methylacidiphilum*, especially since they are clearly functional.

The metabolomic pathway analysis of gene content may have limitations for evolutionary insights in this case. The phylogenetic analysis of the *pmo *genes in this organism in another publication [3] suggested a divergent evolutionary history from methylotrophic proteobacteria, one which might even be consistent with a relatively deep branching core identity for this species, and this line of investigation might be usefully pursued with other genes involved in *Methylacidiphilum *methylotrophy.

*M. infernorum *appears to display gene loss in the cell division system, and what is interpreted as accelerated evolution is claimed to have occurred in the *ftsZ *gene. Considering the non-functional nature of the *ftsZ *homolog in the PVC member *Lentisphaera*, it would be highly relevant for the data and analysis of the data relating to this claim to be described in the paper – what reasons are there for interpretation of presumably divergent sequence as accelerated evolution? Why isn't a deep branching of this and other verrucomicrobial *ftsZ *relative to homologs in other phyla an equally plausible explanation for its divergent sequence? Have long branch attraction artefacts been definitively demonstrated as a most probable alternative explanation for such a deep branch? What is the explanation of accelerated evolution in the apparently retained *ftsZ *but not in *ftsK *and *ftsA*, presumed components of the same *ftsZ*-dependent divisome?

The view that previous speculation interpreted as relating cell division in the PVC superphylum to evolution of the eukaryotic nucleus and cytoskeleton has not so far been supported may be a warranted view at this point. However, such conclusions rejecting PVC superphylum relevance to eukaryote evolution are not only based on limited studies but also on studies based on a very limited sampling of taxa and limited types of bioinformatic analysis. That this may be important is indicated by the occurrence of structures reactive with anti-tubulin antibodies in the 'epixenosome' verrucomicrobial symbionts of ciliate protozoa [83,84] as well as the demonstrated tubulin homologs in *Prosthecobacter *species [41], indicating a potentially wider occurrence of this eukaryote cytoskeletal protein among verrucomicrobia than has been detected and one not as easily explained by lateral gene transfer as by an evolutionary retention of a deep signal. Concerning detection of potentially eukaryote-homologous features within PVC group members, analysis at the level of secondary structure may be needed to reveal unsuspected relationships to eukaryote signature proteins such as nuclear pore complex proteins [85]. Detection of homologs of PVC proteins among eukaryote proteins may be expected to be difficult, since even within eukaryotes detection of important eukaryote signature proteins such as homologs to nuclear pore complexes may not be trivial, since exceptional heterogeneity occurs between species, and BLAST and even PSI-BLAST approaches may fail due to variation in evolutionary rate alone [86]. This question does not appear to be as resolved as the authors have suggested, and relevance of any PVC member to eukaryotes is certainly not refuted by the analysis presented. One might in a lighter mood suggest that it is not over until the sterol-synthesizing nucleated *Gemmata *planctomycete sings.

This paper is important for suggesting and perhaps stimulating a number of lines of investigation for future genomic analysis of the phylum *Verrucomicrobia *and the PVC superphylum, but this future analysis should not be constrained by assumptions concerning easy interpretation of the paradoxes posed by *Methylacidiphilum *and verrucomicrobia, which still seem unsatisfactorily resolved at this point.

#### Authors' response

*We appreciate this detailed, constructive review and completely agree that further phylogenetic analysis, with particular emphasis on detection of HGT, and perhaps, most importantly, sequencing and comparative analysis of additional, diverse representatives of phylum Verrucomicrobia and the PVC superphylum are required before we understand the natural history of this remarkable group of bacteria. Where we tend to be more skeptical than the reviewer, is the possibility of direct relevance of comparative genomics of the PVC superphylum for the origin of the eukaryotic nucleus. The results with anti-tubulin antibodies reported in references 83 and 84 should be treated with utmost caution. Furthermore, we strongly believe that HGT from eukaryotes is the only viable explanation for the presence of tubulins in Prosthecobacteria*[41]. *The difficulty of detecting homologs of nuclear pore complex subunits should not be exaggerated; at least, finding orthologs throughout the eukaryotic diversity was a straightforward (if not, exactly, trivial) task*[47]*. Although one cannot rule out surprises from new genomes, the chances that any representatives of the PVC superphylum actually possess structures that are homologous to the eukaryotic nucleus (and, in particular, the nuclear pore) are exceedingly small. It is another matter that some members of the PVC superphylum can provide extremely interesting and valuable instances of independent, convergent evolution of intracellular compartmentalization.*

#### Reviewer's response in a second review

I welcome the authors' agreement with my comments concerning the need for more analysis of the hypothesized HGT and for further sequencing and comparative analysis of additional representatives of the verrucomicrobia and the PVC superphylum. My only comment on the doubts of even the possibility of direct relevance of comparative genomics of the PVC superphylum for the origin of the eukaryotic nucleus is that absence of evidence does not constitute evidence of absence and that beliefs, however strongly held, are not refutations (e.g. the belief that HGT from eukaryotes is the only viable explanation for *Prosthecobacter *tubulins). This is perhaps especially so considering that gene annotation and decision regarding HGT appears to be a work in progress where no one annotation effort, especially if automated only, can be assumed complete. Chances that are estimated qualitatively to be exceedingly small concerning surprising structures within PVC superphylum members can nevertheless be finite, and prediction of absence of such structures cannot be made with certainty. Improved analysis as well as more genome data may be needed to solve these problems.

##### Authors' response

We agree.

### Reviewer's report 2

Ludmila Chistoserdova, Department of Chemical Engineering, University of Washington, Box 355014, Seattle, WA 98195-5014, USA (nominated by Janet Siefert, Rice University, Houston, TX, United States)

This paper describes analysis of the genome of strain V4, an acidophilic methanotroph belonging to *Verrucomicrobia*. Not only this is the first genome representing this phylum to be formally described, but this is also a rare precedent of a very fast progress from the isolation and description of a novel strain to genome sequencing and analysis, all within one year! This is very exciting.

#### Major comments

##### Methylotrophy

As my main expertise is in methylotrophy, I will concentrate my criticism on the parts of the paper that relate to this area. My main problem with metabolic reconstruction of methylotrophy in strain V4 is realization that the authors presumed that methylotrophy in *Verrucomicrobia *must follow the scheme utilized by *Proteobacteria*, and more specifically by *Methylococcus capsulatus*, a carryover from the original publication [3]. This presumption is first stated on page 8. Quote: 'The abundance of proteins with the greatest similarity to homologs from *Proteobacteria *is compatible with the dominance of the bacterial phylum among methylotrophs'. This does not make any sense, even if by 'bacterial' it is actually meant 'proteobacterial'. Of course, methylotrophy in *Proteobacteria *is most well studied, but with 99% of microbes remaining uncultivated, we do not really know methylotrophs of which group are most abundant in the environment (it could be *Verrucomicrobia*?), or which metabolic scheme(s) they employ. Even within known Bacteria, multiple modes of methylotrophy are recognized, not just the ones operating in *M. capsulatus *or other mainstream laboratory models. As the authors correctly point out, the large proportion of proteobacterial top hits must be due to over-representation of proteobacterial genomes and under-representation of verrucomicrobial genomes in the datasets they used.

##### Authors' response

*In the quoted sentence 'methylotrophs' was corrected to 'the known methylotrophs', and of course, "the bacterial phylum" was replaced with "this bacterial phylum" (we appreciate the reviewer pointing out this unfortunate mistake). Otherwise, however, the meaning appears to be sensible, implying the high likelihood of HGT in both directions. Furthermore, although we indeed do not know the actual taxonomic spread of methanotrophy, the majority of isolated methanotrophs, as well as the most abundant methylotrophs in metagenomic samples analyzed to date *[87,88]*, do belong to Proteobacteria, suggesting that the dominance of this phylum among methylotrophs in the current databases reflects the actual distribution in the biosphere.*

Indeed, like the previously described methanotrophs, V4 appears to encode methane monooxygenase, possessing three gene clusters highly similar to the *pmoCAB *clusters in proteobacteria (up to 60% amino acid identity). However, this may be the only common step in methylotrophy that V4 and methanotrophs such as *M. capsulatus *share (note that formate dehydrogenases are present in all life). One obvious problem with metabolic reconstruction downstream of methanol is the lack of a recognizable methanol dehydrogenase. There are many lines of evidence indicating that the *xoxFJG *gene cluster does not encode a functional methanol dehydrogenase, as follows. (1) These genes are ubiquitously present in both methylotrophic and non-methylotrophic *Proteobacteria*. (2) These genes have been mutated in four methylotrophs that utilize methanol as a sole source of carbon and energy: *Paracoccus denitrificans *[89]*, Methylobacterium extorquens *[90]*, Methylibium petroleiphilum*, and an unclassified *Burkholderiales *strain [91]. Neither of these mutations resulted in a loss of methanol dehydrogenase activity. (3) In *M. petroleiphilum *that does not encode the traditional methanol dehydrogenase (MxaFI), an alternative methanol dehydrogenase (Mdh2) has been identified, and mutating the corresponding gene lead to a methanol-negative phenotype and the loss of methanol dehydrogenase activity [91]. Likely, other methylotrophs not possessing *mxaI *and other essential *mxa *genes encode alternative methanol dehydrogenases or other types of enzymes. (4) In mutants of *M. extorquens *lacking true (MxaFI) methanol dehydrogenase but over-expressing *xoxFJG*, no methanol dehydrogenase activity could be measured [90]. (5) The recent work is quoted in which the Xox system has been implicated in 'formaldehyde metabolism' [33]. The evidence presented in ref. [33] is however so circumstantial that even the authors of this work never claimed that XoxFJG encoded a methanol dehydrogenase. Note the lack of methanol dehydrogenase activity and the fact that XoxFJG appear to be specifically involved in photosynthetic metabolism in *Rhodobacter *while in V4, C_1 _metabolism is not connected to photosynthesis. Based on this knowledge, I would argue that XoxFJG are not responsible for methanol oxidation in V4. What enzyme is responsible then? Not having access to the complete genome sequence I cannot tell, but other candidates should be considered. Note that methanol must not necessarily be processed by a pyrroloquinoline quinone dehydrogenase. The enzyme in question could be a NAD-linked dehydrogenase (used by high GC Gram-positive methylotrophs), it could be an oxidase (used by methylotrophic yeasts), or, alternatively, it could be a methyltransferase (used by methylotrophic Archaea and also by methylotrophic *Clostridia*).

##### Authors' response

*To the best of our knowledge, most of the characterized methanol dehydrogenases belong to a specific family of PQQ-dependent dehydrogenases, methanol/ethanol family (COG4993 in the COG database, *[13]*). It is true that this family includes enzymes with other substrate specificities. In most methylotrophs with large genomes, there are several enzymes of this family, and if they can substitute each other, this could explain the retention of the methanol dehydrogenase activity when some of them are mutated. Furthermore, these enzymes might have a wide specificity spectrum. The genome of M. infernorum encodes only one protein from this family (Minf_0992). Other potential activities of this enzyme, i.e. glucose dehydrogenase or alcohol dehydrogenase, seem unlikely: glucose dehydrogenase is rarely found in autotrophic organisms, and there are better candidates (e.g., Minf_0269, Minf_1850) for the classic alcohol dehydrogenase function. As for potential alternative enzymes to catalyze this step, that does not seem likely. The M. infernorum genome does not encode close homologs of either archaeal/clostridial methanol:corrinoid methyltransferase MtaB *[92]* or NAD-linked methanol dehydrogenase (member of the iron-containing alcohol dehydrogenase family, COG1454), found in Bacillus methanolicus and related bacteria *[93]*. Although M. infernorum does encode a predicted flavoprotein (Minf_1595) that is distantly related to the FAD-dependent methanol oxidase of methylotrophic yeasts *[94]*, the low sequence similarity strongly suggests that Minf_1595 has a different function. All these alternative methanol dehydrogenases have complex phylogenetic distributions, for example, a close homolog of MtaB is encoded in the unfinished genome of Opitutaceae bacterium TAV2, another member of Verrucomicrobia. Thus, for M. infernorum, the best candidate for methanol dehydrogenase is Minf_0992, the only PQQ-dependent enzyme encoded in its genome.*

The second problem with reconstructing methylotrophy, as proposed, is the lack of any of the major systems for formaldehyde oxidation that have been proven essential (i.e. the tetrahydromethanopterin-linked pathway, the glutathione-linked pathway, the oxidative branch of the ribulose monophosphate cycle, or the specific NAD-linked formaldehyde dehydrogenase). Discussion of this problem is avoided in this manuscript. However, in the original paper [3], as well as in Fig. 5 of this manuscript, the function of formaldehyde oxidation is casually ascribed to some unidentified alcohol dehydrogenase(s). It is not so. Few alcohol or aldehyde dehydrogenases actually express affinity for formaldehyde, so it is very unlikely that non-specific dehydrogenases could account for efficient formaldehyde oxidation.

##### Authors' response

*We agree, there is no solid candidate for this function. We have changed Figure *5* to show this reaction by a dashed line. We do expect the enzyme for this step to be present in the M. infernorum genome but do not have sufficient evidence to suggest a good candidate.*

On another hand, *folD *and *ftfL *are present in the genome of V4, but these genes are ubiquitous, and they are typically assumed to be involved in various essential biosynthetic processes, such as purine and thymidylate syntheses. However, involvement of FolD in methylotrophy has been demonstrated before, at least in one case [95,96]. Specifically, FolD, in combination with MetF and PurU were shown to be involved in metabolism of chloromethane in *Methylobacterium chloromethanicum*. Interestingly, this pathway is not involved in oxidation of methanol by *M. chloromethanicum*. I think, the presence of *folD *and *ftfL *and the absence of any other systems for handling formaldehyde deserve a more thorough discussion.

##### Authors' response

*We agree. The proposed involvement of FolD (Minf_0806) and FtfL (Minf_0373) in formaldehyde metabolism (Fig. *5*) is just a conjecture that needs to be verified experimentally. The quoted references have been included in the main text.*

Very puzzling to me was the attempt to imply that elements of methylamine dehydrogenase were present, given the fact that the organism does not grow on methylamine. An 'amine dehydrogenase' may potentially be encoded, with an unknown substrate specificity, but there is no evidence, with a number of essential gene homologs missing, that the putative enzyme system would be a methylamine dehydrogenase.

##### Authors' response

*The phrase in question has been modified. M. infernorum genome contains a 5-gene operon (mauBEDAmoxG. Minf_1997–2001), which encodes proteins that are strongly similar to the subunits of methylamine dehydrogenase (MauB and MauA), methylamine utilization proteins MauE and MauD and cytochrome c from methylamine-utilizing bacteria *[36]*. A MauG homolog (Minf_1905) is encoded elsewhere in the genome. There is no obvious reason to suggest that these genes are not functional or have any other specificity. However, we do note in the paper that attempts to grow M. infernorum V4 on methylamine have been unsuccessful, which could be due, e.g., to the absence of amicyanin, or accessory proteins MauF and MauL. Nevertheless, there remains a possibility that methylamine dehydrogenase could be assembled in M. infernorum in the absence of those missing genes and that, under certain growth conditions, M. infernorum would be able to utilize methylamine.*

Attempts at reconstructing assimilatory C_1 _metabolism were equally puzzling. When it comes to C_1 _assimilation, three well-characterized modes are known (described in detail by Anthony [38]). Two of these involve assimilation at the level of formaldehyde (the serine and the ribulose monophosphate cycles) and one involves assimilation at the level of CO_2_, via the CBB (Calvin) cycle. The genome of V4 appears to have all the genes to code for the latter, but it offers very little evidence for operation of the serine cycle. Genes claimed to encode serine glyoxylate aminotransferase and hydroxypyruvate reductase actually encode polypeptides with less than 30% identity to the respective enzymes with a proven function. At this level of similarity and without any experimental evidence, these should be classified as 'an aminotransferase family protein' and 'a putative hydroxyacid dehydrogenase family protein', respectively. *glyA *was identified with higher confidence, but this gene is not indicative of methylotrophy, as this gene is ubiquitous in nature. The two key enzymes of the serine cycle, encoding malyl-CoA lyase and glycerate kinase are missing. Their absence does not suggest alternative routes to me, but it rather suggests that the serine cycle is not encoded, and points to the importance of the CBB cycle. Operation of the energy-demanding CBB cycle also agrees with the low biomass yields observed for V4 when grown on methane.

##### Authors' response

*There is little doubt that M. infernorum is capable of assimilating CO_2 _via the Calvin cycle and lacks the RuMP pathway. The question therefore is whether all utilized methane has to be oxidized to the CO_2 _level and then re-reduced in the course of the Calvin cycle. Obviously, direct utilization of methylene groups, as proposed in Fig. *5*, is a far less wasteful and therefore a more parsimonious pathway. Reconstruction of the serine cycle as the potential main route for C_1 _assimilation was based on the presence of good candidates for all enzymes except for glycerate kinase. Given the existence of three entirely different classes of glycerate kinases *[97]*, it is not much of a stretch to propose the existence of yet another variant of that enzyme. Malyl-CoA lyase is not needed in our reconstruction because the glyoxylate shunt is present and provides glyoxylate for the proposed serine cycle. Nevertheless, we realize that Figure *5* offers only a tentative reconstruction of M. infernorum metabolism. We hope that this scheme provides a plausible direction for the further experiments which will prove or disprove our hypothesis.*

I suggest that the reconstruction of methylotrophy be streamlined as follows: methane is oxidized by one of, or by all three of the PmoABC systems. The resulting methanol is converted to either formaldehyde by a (non-PQQ) dehydrogenase or by an oxidase, or to methyl-H_4_F by a methyltransferase (whichever could be identified in the genome with higher confidence). In the first scenario, formaldehyde would have to condense with H_4_F non-enzymatically to produce methylene-H_4_F. The latter is then oxidized all the way to CO_2_, and CO_2 _is assimilated via the CBB (Calvin) cycle. Fig. 5 should be streamlined accordingly: methylamine removed, non-specific formaldehyde dehydrogenase removed, the serine cycle and the fantasy connections to other pathways removed.

##### Authors' response

*We have addressed the concerns about the methanol dehydrogenase above and have every reason to stick to our original reconstruction here. As for serine cycle, our hypothetical scheme (Figure *5*) relies on the presence of the corresponding genes in the M. infernorum genome. We are not aware of any organism that would oxidize methane to CO_2 _and then use CO_2 _as the sole carbon source. Of course, the proposed pathway remains to be verified (or falsified) in direct biochemical experiments.*

Also in this Figure: the MetF reaction is incorrect. MetF reversibly oxidizes methyl-H_4_F into methylene-H_4_F. The glyoxylate shunt is shown incorrectly. Isocitrate lyase (AceA) cleaves isocitrate into succinate and glyoxylate. Malate synthase (AceB) condenses glyoxylate with acetyl-CoA to produce malate. All three *pmo *genes (ABC) should be indicated, as methane monooxygenase has three subunits.

##### Authors' response

*Figure *5* has been modified as suggested.*

##### Comparisons with other *Verrucomicrobia*

It is absolutely necessary to re-run BLAST analyses with the newly available *Verrucomicrobia *genomes (*Opitutus terrae*, Bacterium Ellin514 and *Verrucomicrobium spinosum*). I predict that comparisons with these genomes may significantly affect some statistics, such as the number of predicted proteins with no homologs (page 6) and the percent of top hits within and outside of the phylum (page 7 and Fig. 3). I expect that data on the abundance of proteins with top hits with homologs in *Proteobacteria *will significantly change. If not, it would make for a better argument in favor of lateral transfers from *Proteobacteria*. For the same reasons, Correspondence Analysis should also be re-done to include these new verrucomicrobial genomes.

##### Authors' response

*For the revised version of the manuscript, BLAST analyses have been performed with the latest version of the NCBI RefSeq protein database (as of May 28, 2008) which, in addition to the unfinished proteome of Opitutaceae bacterium TAV2 (4036 proteins), included protein sets encoded in the complete genomes of Opitutus terrae (4612 proteins) and Akkermansia muciniphila (2138 proteins), as well as in unfinished genomes of bacterium Ellin514 (6402 proteins) and Verrucomicrobium spinosum (6509 proteins). An increase in the number of verrucomicrobial proteins in RefSeq from 4,036 to 23,697 increased the fraction of best BLAST hits from M. infernorum into the Chlamydia/Verrucomicrobia lineage from 12% to 46% but did not dramatically change the relative contribution of other phylogenetic groups. For example, the fraction of best hits into Proteobacteria decreased from 51% to 30% but they still remained the largest target group outside of Chlamydia/Verrucomicrobia (Supplementary Figure 7 [see Additional file *2*]). For consistency, other analyses that had been performed on the original representative set of 59 genomes (Supplementary Table 1 [see Additional file *1*]) were kept unchanged.*

##### Comparisons with other methylotrophs

It has been stated a number of times that the genome described here is a smaller genome compared to other methylotrophs (for example, page 12, first paragraph), and in this light, the genome streamlining strategies are described. However, the existence of a much smaller methylotroph genome, of *Methylophilales *HTCC2181 [34], that is actually the smallest genome so far for a free-living bacterium (1.3 Mb), has been ignored. It is important, at least in terms of genome streamlining, to compare these two genomes.

##### Authors' response

*A detailed analysis of the genome of Methylophilales bacterium HTCC2181 (GenBank accession number *AAUX01000000*), while obviously a very interesting project, was outside the scope of this study, which dealt primarily with finished (completely sequenced) genomes. In this paper, streamlining of the M. infernorum genome is mostly envisaged in its taxonomic context within the Verrucomicrobia/Chlamydiae and Planctomycetes group (Supplementary Figure 3 [see Additional file *1*]) and in comparison with completely sequenced methylotrophic genomes (Supplementary Figures 4–5 [see Additional file *1*]). A detailed study of genome streamlining in various methylotrophs would be more appropriate in the near future, after additional complete genomes of methano- and methylotrophs from different taxa become available to the public. We did not feel comfortable analyzing in detail an unfinished genome that was sequenced by others.*

##### Statistics

In Table 1, the number of CDSs is different from the number of protein coding genes. I think both need definitions. The numbers still do not add up. 1,612 proteins are included into the analysis and 731 are mentioned not to have homologs, this makes only 2,343, versus 2,478 CDSs and versus 2,474 protein coding genes.

##### Authors' response

*The numbers have been corrected according to the GenBank submission (*CP000975*), some terms used in Table *1* have been clarified.*

In Figure 3, it is not explained that apparently only proteins with 'hits' were included in the analysis. To improve Fig. 3, all the proteins should be included. It also needs to be stated what 'no hit' means, what was the threshold for calling gene/protein homologs, and what the 'unresolved' in Fig. 3 stands for.

##### Authors' response

*The legend to Figure *3* has been updated and a detailed description of the analysis procedure has been included into the Methods section.*

##### Organism's name

The authors should state their intent to publish a formal description of strain V4, and the timeline for that if any. At this time, three groups cultivating methanotrophic *Verrucomicrobia *refer to them as three different organisms while they appear to represent a single species, based on their close relatedness at the 16S rRNA gene level. It would be unfortunate and confusing if all three *candidatus *names make it into the literature. In this light, it is OK to mention the *candidatus *name preferred by the authors once or twice, but it is best to refer to the organism as 'strain' or 'isolate' V4 throughout the text.

##### Authors' response

*The three groups that independently isolated methanotrophic Verrucomicrobia *[3-5]* have agreed to provide a joint description of the three isolates, including strain V4, proposed collectively to represent the genus 'Methylacidiphilum'. A joint manuscript is currently in preparation. Prior to submission, these strains will be deposited in two internationally recognized culture collections as required by the International Committee on Systematics of Prokaryotes.*

##### Minor comments

Page 10, line 5 from bottom, did you mean housekeeping?

##### Authors' response

*No, Maf is indeed a house-cleaning protein *[98].

Page 15, line 3, you meant methanol, right?

##### Authors' response

Indeed, corrected.

Page 15, same paragraph. Note that many pyrroloquinoline quinone dehydrogenases (such as glucose-, ethanol-, butanol dehydrogenases *etc*. require PQQ as a cofactor).

##### Authors' response

The predicted methanol dehydrogenase is the only enzyme from this family encoded in the M. infernorum genome.

Page 15, lines 6–9 from the bottom. Ambiguous sentence. Of course *Methylocella *and *Methylocapsa *use the serine cycle and not the ribulose monophosphate cycle, because they are Type II methanotrophs. This group also includes *Metylosinus *and *Methylocystis*. Reference 7 could not have mentioned *Methylocella *or *Methylocapsa *as they have not yet been discovered in 1996.

##### Authors' response

Indeed, corrected.

Page16, line 2. *M. extorquens *is not an obligate methylotroph, it is a classic example of a facultative methylotroph.

##### Authors' response

Indeed, corrected.

Page 17, second paragraph. Formate dehydrogenases should not be referred to as pathways. Also note, line 3, that formate dehydrogenases are present in all known organisms, not just methylotrophs.

##### Authors' response

Indeed, corrected. 'Formate' has been changed to 'formaldehyde'

Page 20 and Table 1, the description of CRISPR is not clear, describe how the 25 repeats are organized in 6 clusters, even better provide a supplementary figure showing CRISPR along with *cas *genes and Minf_0882. Is it true that CRISPR presence is typical of thermophiles? I thought they were widespread across temperature optima. Explain what you mean by thermophilic determinant: this gene is not present in CRISPR-containing genomes of mesophiles? Then you need to explain that CRISPR systems are also typical of mesophiles.

##### Authors' response

*The CRISPR system is only tangentially relevant to the general description of the M. infernorum genome and its most interesting features such as methanotrophy and adaptation to acidic environment. Given our observation that the CRISPR system might not be fully functional, we do not feel the need to provide an additional figure. Regarding the thermophilic determinants, we provide a brief definition and cite an earlier paper *[55]* where this has been discussed in detail.*

Page 20, last paragraph. The conclusion on 'elaborate system of stress response' does not seem justified as no description of stress response systems is given, except for a potentially non-functioning CRISPR-based anti-virus defense and the putative toxin-antitoxin system (why should V4 worry about toxins?).

##### Authors' response

The phrase in question has been reformulated.

Are there any stress-response systems associated with the extreme ecological niche the organism inhabits?

##### Authors' response

*We discuss possible adaptations to the acidic environment and cite several relevant papers *[49-51].

### Reviewer's report 3

Radhey S. Gupta, Department of Biochemistry and Biomedical Sciences, McMaster University, Hamilton, Ontario L8N 3Z5, Canada (nominated by Jonathan Eisen, University of California Davis Genome Center, Davis, California, USA)

In this manuscript Hou et al. report the genome sequence for "*Candidatus *Methylacidiphilum infernorum", which is the first representative from the phylum *Verrucomicrobia *to be completely sequenced. The organisms from this clade of bacteria are in general very poorly characterized and only few of them have been isolated in pure culture. Hence, the availability of genome sequence for a member of this group should prove very useful for a variety of studies, particularly in clarifying its phylogenetic placement relative to other bacterial phyla. The authors have carried out detailed analyses of *M. infernorum *genome to reconstruct its central metabolic pathway and have identified many genes/proteins responsible for its ability to utilize methane and adaptation to the acidic environment. Several differences were noted from other methylotrophs, which are mainly alpha- and gamma-proteobacteria. The work on the annotation of various genes involved in different cellular functions and the differences seen in these regards for *M. infernorum *has been competently carried out and I have no questions or concerns.

Another important aspect of this manuscript relates to the phylogenetic placement of *Verrucomicrobia *with respect to other bacterial phyla. Recent studies by a number of authors, primarily based on 16S and 23S rRNA [1,2,48], have indicated that species from three bacterial phyla viz.*Planctomycetes, Verrucomicrobia-Lentisphaerae *and *Chlamydiae *(as well as *Poribacteria *and OP3), group together in phylogenetic trees. This has led to the proposal that these groups or phyla should be recognized as part of a single superphylum (PVC). In this work the authors have constructed phylogenetic trees based on concatenated sequences for 51 ribosomal proteins and also the three subunits for RNAP. The trees based on these sequences also support the grouping of these species in a single clade. Other analyses reported here to determine the closest relatives of *M. infernorum *(e.g. top BLAST hits, Correspondence analysis) have provided no clarification in these regards and these results have been interpreted to suggest a complex evolutionary history of the *Verrucomicrobia*.

#### Main Comments

1. The proposal that the PVC group of species should be recognized as a superphylum is presently entirely based on some phylogenies. As noted by the authors, other phylogenetic studies have not always supported this grouping. In our recent work [18], phylogenetic analysis was carried out based on concatenated sequences for 11 large and conserved proteins (including alpha, beta and beta' RNAP). Although these analyses strongly supported the grouping of *Chlamydiae *and *Verrucomicrobia*, a reliable grouping of the *Planctomycetes *with these groups was only observed in the neighbour-joining tree, but not supported by the maximum-likelihood analysis. Thus, based upon the phylogenetic analyses alone, it is difficult to infer confidently whether the PVC groups of species should be recognized as a superphylum or not. Because, the term Superphylum has taxonomic significance, this status should not be accorded readily unless different lines of evidences support the inference. In the present work, the authors have looked for but failed to find any signature protein that was unique to the PVC groups of species. The closest they have come to identifying a signature protein for this group is the Minf_1886 protein that is only found in *Planctomycetes *and *Verrucomicrobia*, but not in any of the chlamydiae.

2. The question thus arises whether the placement of PVC groups of species into a single superphylum is supported by any other line of evidence besides some of the phylogenetic trees. Interestingly, contrary to the authors' observation that they did not find any protein that was unique to the PVC groups of species, we have identified a protein CT421.2 that is uniquely present in all available sequences from the *Planctomycetes, Verrucomicrobia-Lentisphaerae *and *Chlamydiae *phyla. The sequence alignment of this protein is presented in Supplementary Figure 8 [see Additional file 2]. A large number of positions in this small protein are highly/completely conserved in all of the species. Besides, the PVC group of species, no other BLAST hit for this protein was observed. The identification of this signature protein that is unique for the PVC groups of species provides additional independent evidence for their grouping into a Superphylum. Sequence information for *M. infernorum *was not available to us, but we expect that this protein should be present there as well. It is also of interest to note that within this protein, a 2 aa indel is present in various *Chlamydiae *species, but not in any of the *Planctomycetes *or *Verrucomicrobia-Lentisphaerae *species. This indel is indicated to be an insert in the *Chlamydiae *species rather than a deletion in the other groups (see next comment). It is unclear why the authors in their analyses did not identify this protein. However, since these results are of importance for the inferences drawn here (and in earlier studies) concerning the PVC superphylum and the authors should discuss their significance in the main manuscript.

##### Authors' response

Indeed, this protein (Minf_0061) is encoded in M. infernorum genome and, as predicted by the reviewer, lacks the 2-aa indel, as do other members of Planctomycetes or Verrucomicrobia-Lentisphaerae species. This sequence has been missed by our automated analysis because of an error of the automated gene calling procedure, which resulted in a truncated 37-aa protein (YP_001938720.1) that missed 13 N-terminal amino acid residues and did not produce sufficiently significant BLAST hits to be recognized in our analysis. We greatly appreciate the reviewer's comment thanks to which the sequence of Minf_0061 has been corrected (YP_001938720.2) and included in the alignment.

Since this manuscript concerns with the phylogeny of *M. infernorum*, it will also be of interest for the author to indicate whether the sequences from this species contain the two conserved inserts in the LysRS and RpoB proteins that were previously reported to be specific for various *Chlamydiae *and *Ver. spinosum *[18]. My updating of the RpoB sequences indicates that the 3 aa insert in this protein is uniquely found in all of the *Chlamydiae, Verrucomicrobia *(*V. spinosum DSM 4136, Opitutus terrae PB90-1, Opitutaceae bacterium TAV2, Akkermansia muciniphila ATCC BAA-835 and Bacterium Ellin514*) and *Lentisphaerae *(*Victivallis vadensis ATCC BAA-548 *and *Lentisphaera araneosa*) species, but not in any other RpoB homologs from different groups of bacteria including the *Planctomycetes *(>400 sequences available in the NCBI database), see in the attached file (Supplementary Figure 9 [see Additional file 2]). The insert in the RpoB protein is thus specific for the *Chlamydiae *and *Verrucomicrobia-Lentisphaerae *phyla and it provides strong and direct evidence that species from these groups shared a common ancestor exclusive of the *Planctomycetes*. This inference is also strongly supported by various phylogenetic trees. Since this work reports the first genome of a *Verrucomicrobia *species, the close relationship that this group exhibits to the *Lentisphaerae *and *Chlamydiae *species (exclusive of the *Planctomycetes*) deserves to be emphasized, apart from the fact that all of these groups are also part of a higher clade (i.e. the PVC clade).

##### Authors' response

*Indeed, RpoB of M. infernorum (Minf_0804) has the same 3-aa insert, and indeed, this indel supports the existence of a separate Chlamydia/Verrucomicrobia clade, as do our results shown in Figure *2* and Supplementary Figure 1 [see Additional file *1*]. However, the Chlamydia/Verrucomicrobia group appears to be widely accepted in the scientific community genome, which is why we did not feel it was necessary to stress this point.*

Other comments:

1. The authors should provide some further details regarding the phylogenetic analyses. They should indicate whether the aligned sequences were edited (if so, how) and how many aligned characters were present in the final two datasets that they have employed. The sequence alignments for these dataset could also be included as supplemental data. It will also be useful if it could be mentioned in the Figure legends that the trees shown are maximum-likelihood trees.

##### Authors' response

*The necessary details on the alignment filtering and the size of the dataset have been added to the text. In the legend to Figure *2*, it is now indicated that Maximum Likelihood trees are shown. Sequence alignments in the aligned FASTA text format have been made available on the FTP site *[99].

2. Page 7, first line, I am not sure whether *Lentisphaerae *is now regarded as a distinct phylum. In the Bergey's manual (2005) *Victivallaceae *is indicated to be a family within the phylum *Verrucomicrobia*.

##### Authors' response

*The suggestion by Cho and colleagues *[100]* that Lentisphaerae forms a distinct phylum has been subsequently validated by Euzéby *[101]*. There seems to be a general agreement that Lentisphaerae should be regarded as a separate phylum *[1,48]*.*

## Supplementary Material

Additional file 1 Supplementary Figures 1–6 and Supplementary Tables 1–3. Click here for file

Additional file 2 Supplementary Figures added in the course of the Open Review. Supplementary Figures 7–9 Click here for file
