# Supplementary material for: Complete genome sequence of the extremely acidophilic methanotroph isolate V4, Methylacidiphilum infernorum, a representative of the bacterial phylum Verrucomicrobia
Source: Biol Direct. 2008 Jul 1;3:26. doi: 10.1186/1745-6150-3-26 (PMC2474590; doi:10.1186/1745-6150-3-26)
Supplement: Additional file 1 — Supplementary Figures 1–6 and Supplementary Tables 1–3. [file 1745-6150-3-26-S1.pdf]

Supplementary Figure 1. Phylogenetic tree based on concatenated subunits of the RNA polymerase

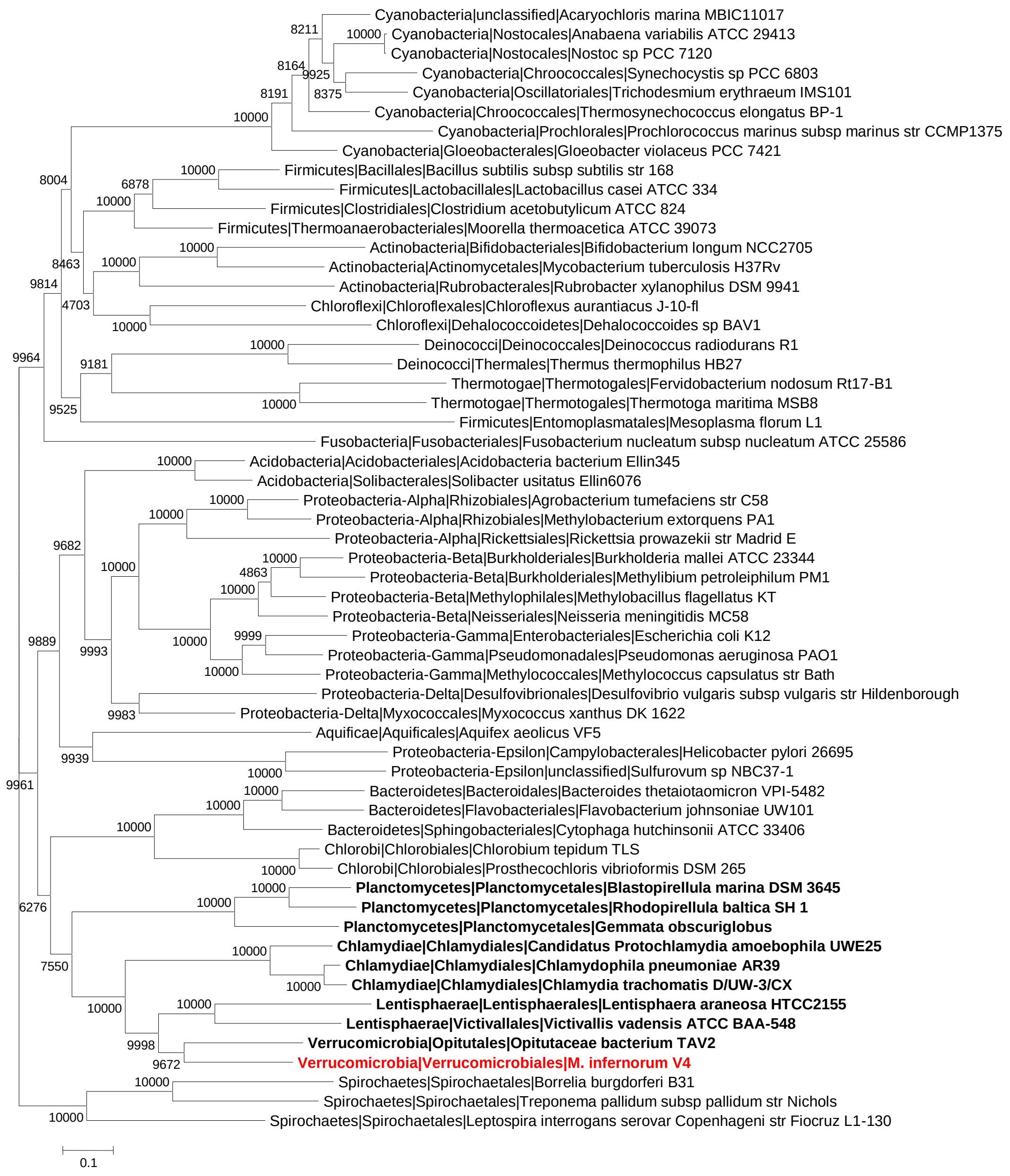

Phylogenetic tree of 59 selected bacteria (listed in the Supplementary Table 1) constructed on the basis of concatenated alignments of three subunits (alpha, beta, beta') of the DNA-dependent RNA polymerase. Bootstrap values are indicated for all branches. Three taxonomy level assignments are separated by the bar symbol. Representatives of the *Planctomycetes/Verrucomicrobia/Chlamydiae/* superphylum are shown in boldface.

Supplementary Figure 2. Multiple alignment of the sole protein family specific for *Planctomyces*/*Verrucomicrobia*/*Chlamydiae* group.

```
149198191|Lenti|Lentisphaera_araneosa_HTCC2155          -MDPDYSLFLNTLNHDHPEWSIEALAFICRGVDFF-----------------------------NSAAPQQ----TT---
168699635|Planc|Gemmata_obscuriglobus_UQM_2246          -----MDPRILELCREDSRFAYEAYEFVSEAVTFTQ-------------------------GRLGRTPADRGD-DGDDR-
150386448|Lenti|Victivallis_vadensis_ATCC_BAA-548       -DQQDVEQKIEELVRRDPRYRHDAYPFVANAVTFT----------------------------VGRLPKHR---------
Minf_1886|Verru|Methylacidiphilum_infernorum            -RKRDFTTIVEEIVEKDSRYLKESYYFVRDGLEYTL-------------------------KTMGKQKHQKIE-------
153892628|Verru|Opitutaceae_bacterium_TAV2              -AELDINQVIDLIRKEDSRYPKTAYLFIREGLDQTV-------------------------KQIKQKDSAR---AQRSL-
32472214|Planc|Rhodopirellula_baltica_SH_1             --MPSPVKAMQDLLRDDTRYKLEAYQFIREALQYAH------------ENLD-----AIGPLGFGPSDDPN---SDAPR-
87308250|Planc|Blastopirellula_marina_DSM_3645          -MTDETYSDFMRMLKEDPRFKLEAYQFVREALSFGQNLFELEDDDDDEEELDLDEEMILDEEEFDDDEEDDIEWSEPER-
166833790|Verru|Akkermansia_muciniphila_ATCC_BAA-835    -TTPTFEDAVSRIVRKDPRFAERAYSFLKDALDFT----------------------------MQRVEERE---NGSQR-
91201105|Planc|Candidatus_Kuenenia_stuttgartiensis      --MKNPWNKIKEIEKKDPRYSPMAYQFVFEALEFTT-------------------------NMLGKTQYKA---ADSGR-
149173500|Planc|Planctomyces_maris_DSM_8797             --MGEKMTSATNLSRPKLQFHPNAYDFIFEALQQAQ-------------------------EIFSRQATQE---DEQEEA


149198191|Lenti|Lentisphaera_araneosa_HTCC2155          DSSAQDLIHSLCLHALNEYGPLALHTLNGLNFHKISDLIQVINLLIERKIITGIERFELENRTLNQSLCTKFPEKSLPEPH
168699635|Planc|Gemmata_obscuriglobus_UQM_2246          HVSGAELLNGACELAVREFGMMAPVVFKQWGLKTTDHVGEIVFKLIKVKLLSKSDRDDPVDFSDLFDLHEALRNGFELTLR
150386448|Lenti|Victivallis_vadensis_ATCC_BAA-548       HVSALELLHGVRDYAQKEFGVLAQQVLDEWGIHEAADVGSIVYSLIGAELLSASQDDSPEEFEIDFPL-------IEPESG
Minf_1886|Verru|Methylacidiphilum_infernorum            QLSGKQILEGLKDYALKEFGPMSKLVLNEWGVKSCRDFGQMIKNMDLYGLLGKTDMGDMKDFQKGYSFTAAFVKPFLPIRS
153892628|Verru|Opitutaceae_bacterium_TAV2              HVSGRELSEGLRHFALDRFGPMAKTVLNEWGIRKSDDFGEIVYNLIDYNVFSKTDTDRREDFASVFDFEEAFVKPFLPACR
32472214|Planc|Rhodopirellula_baltica_SH_1             HLTGQQLCEACRLYAIDQFGYLAQMVLENWGLRRTGDFGELVYNLIRIEQMRKSESDRREDFDDVYDFENAFQPKFELALS
87308250|Planc|Blastopirellula_marina_DSM_3645          HLTGQMLCEAIRNFAQQQYGLMAKTVLNSWGIETTGHFGDIVYNLIEIGMMKKSPQDRREDFDDVYDFDAAFVRDFRIEVS
166833790|Verru|Akkermansia_muciniphila_ATCC_BAA-835    HVSGQELLEGFRDYALAQFGPMASTVMKEWGLRNGKNVGEMVFLLIEEDVFSKQPEDSLDDFKGFMSFRKAFEEPYEFQDQ
91201105|Planc|Candidatus_Kuenenia_stuttgartiensis      HVTGKELLEGIKRYALKQFGFMALTVFEQWGIKEDIDFGNIVFNLVESGLMGKTETDSKDDFKNIYNFKKVFDEEYEFKGN
149173500|Planc|Planctomyces_maris_DSM_8797             HVSGQELLEGVRELALKQFGLMTLTVFKQWGVQSTRDFGKMVFEMIEHGRMRKTEHDRLEDFVDIYDFEQVFDVAYVIDTS
```

Multiple alignment was constructed using the MUSCLE program (Edgar RC: **MUSCLE: a multiple sequence alignment method with reduced time and space complexity**. *BMC Bioinformatics* 2004, **5**:113). For each sequence the GI (gene identification) number (or locus identifier in case of *M. infernorum* V4), abbreviated phylum name, and the complete organism name are indicated. Phylum abbreviations are the as follows: Lenti - *Lentisphaerae*; Planc - *Planctomycetes*; Verru - *Verrucomicrobia*;

Supplementary Figure 3. Functional distribution of gene gain and loss as reconstructed by comparison with Last Common Ancestor of Bacteria (LBCA).

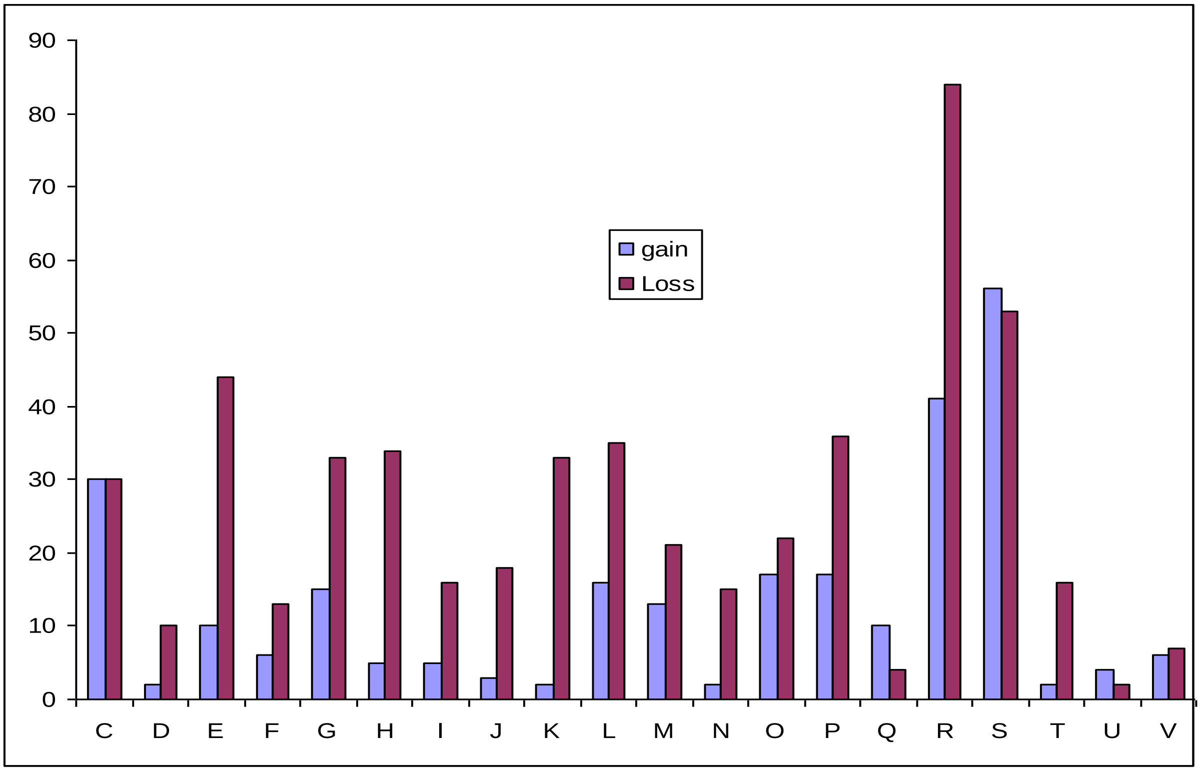

The number of genes in each category is shown on the vertical axis. Designations of functional groups (from the COG database) on the horizontal axis are as follows: J – Translation, ribosomal structure and biogenesis; K – Transcription; L – DNA replication, recombination and repair; D – Cell division and chromosome partitioning; O – Posttranslational modification, protein turnover, chaperones; M – Cell envelope and outer membrane biogenesis; N – Cell motility and secretion; P – Inorganic ion transport and metabolism; T – Signal transduction mechanisms; C –Energy production and conversion; G – Carbohydrate transport and metabolism; E – Amino acid transport and metabolism; F – Nucleotide transport and metabolism; H – Coenzyme metabolism; I – Lipid metabolism; Q – Secondary metabolites biosynthesis, transport and catabolism; V – genes involved in stress response and microbial defense.

Supplementary Figure 4. Transporter genes in bacterial genomes.

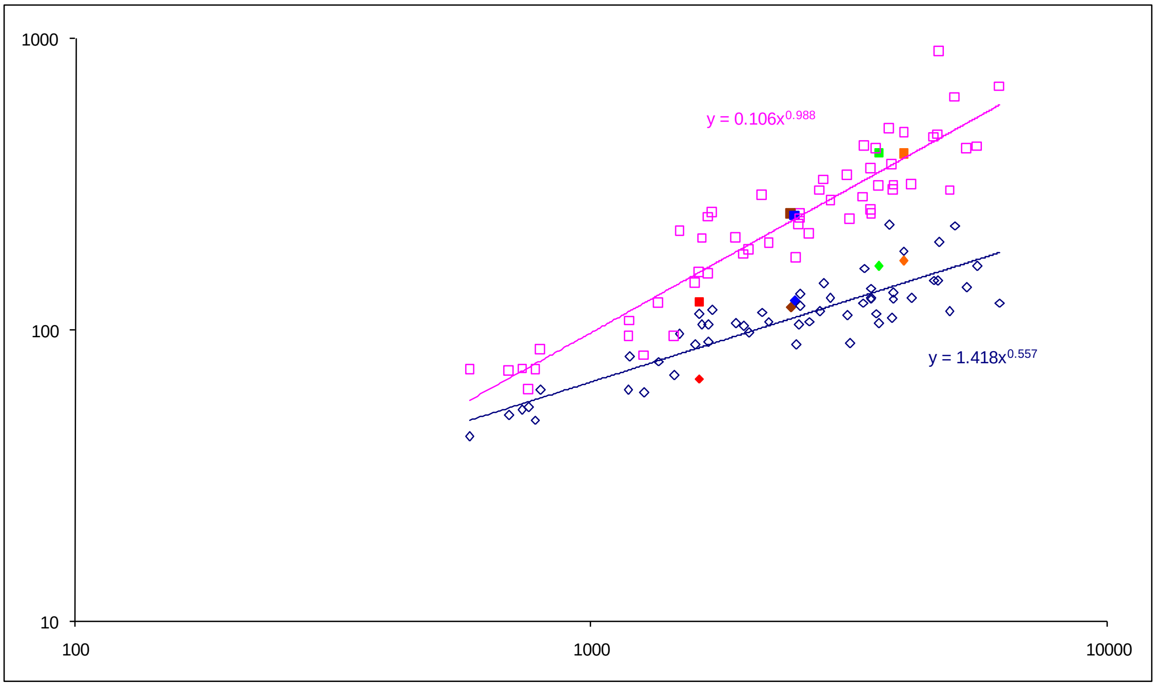

Vertical axis – the number of transporters (squares) and the number of distinct transporter families (COGs, diamonds). Horizontal axis – the genome size (the number of encoded proteins).

Red - *M. infernorum* V4; brown - *Methylobacillus flagellatus* KT; green - *Methylibium petroleiphilum* PM1; light blue - *Methylococcus capsulatus* str. Bath; orange - *Methylobacterium extorquens* PA1.

Supplementary Figure 5. Transcriptional regulators in bacterial genomes.

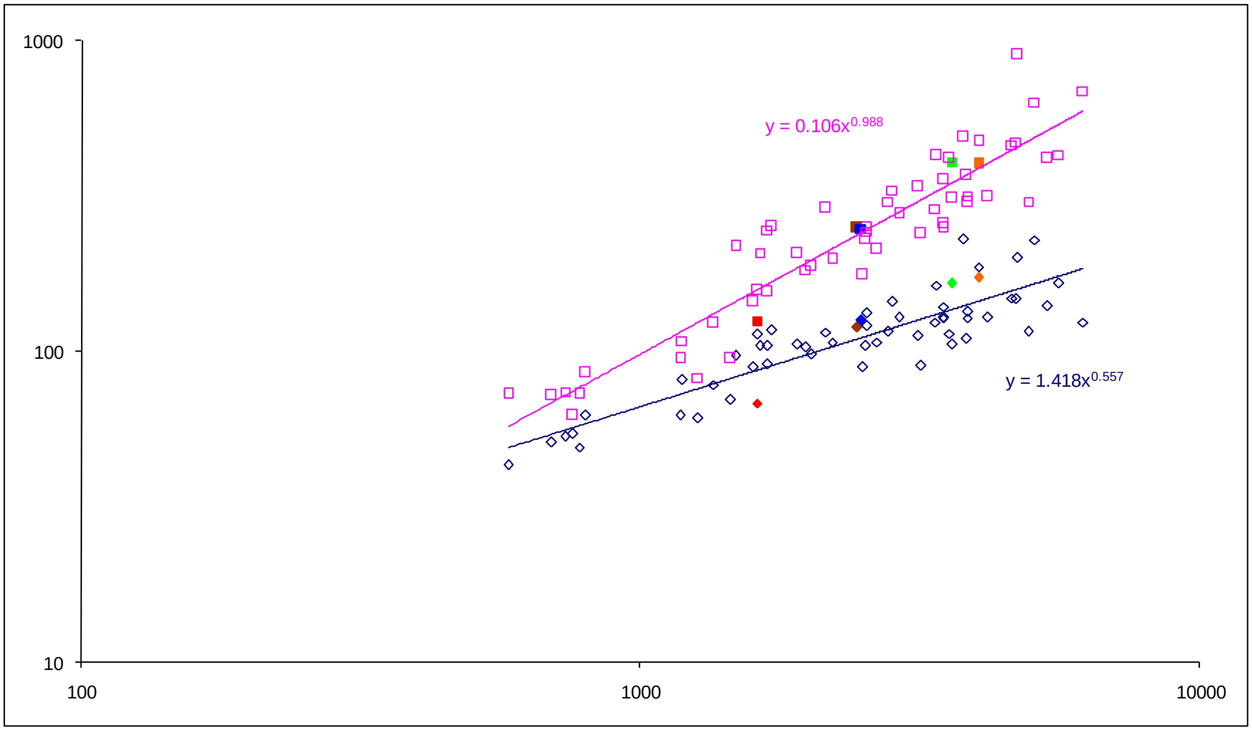

Vertical axis – the number of transporters (squares) and the number of distinct families of transcriptional regulators (COGs, diamonds). Horizontal axis – the genome size (number of encoded proteins).

Red - *M. infernorum* V4; brown - *Methylobacillus flagellatus* KT; green - *Methylibium petroleiphilum* PM1; light blue - *Methylococcus capsulatus* str. Bath; orange - *Methylobacterium extorquens* PA1.

Supplementary Figure 6. Phylogenetic tree of beta (catalytic) subunits of H^+-ATPases

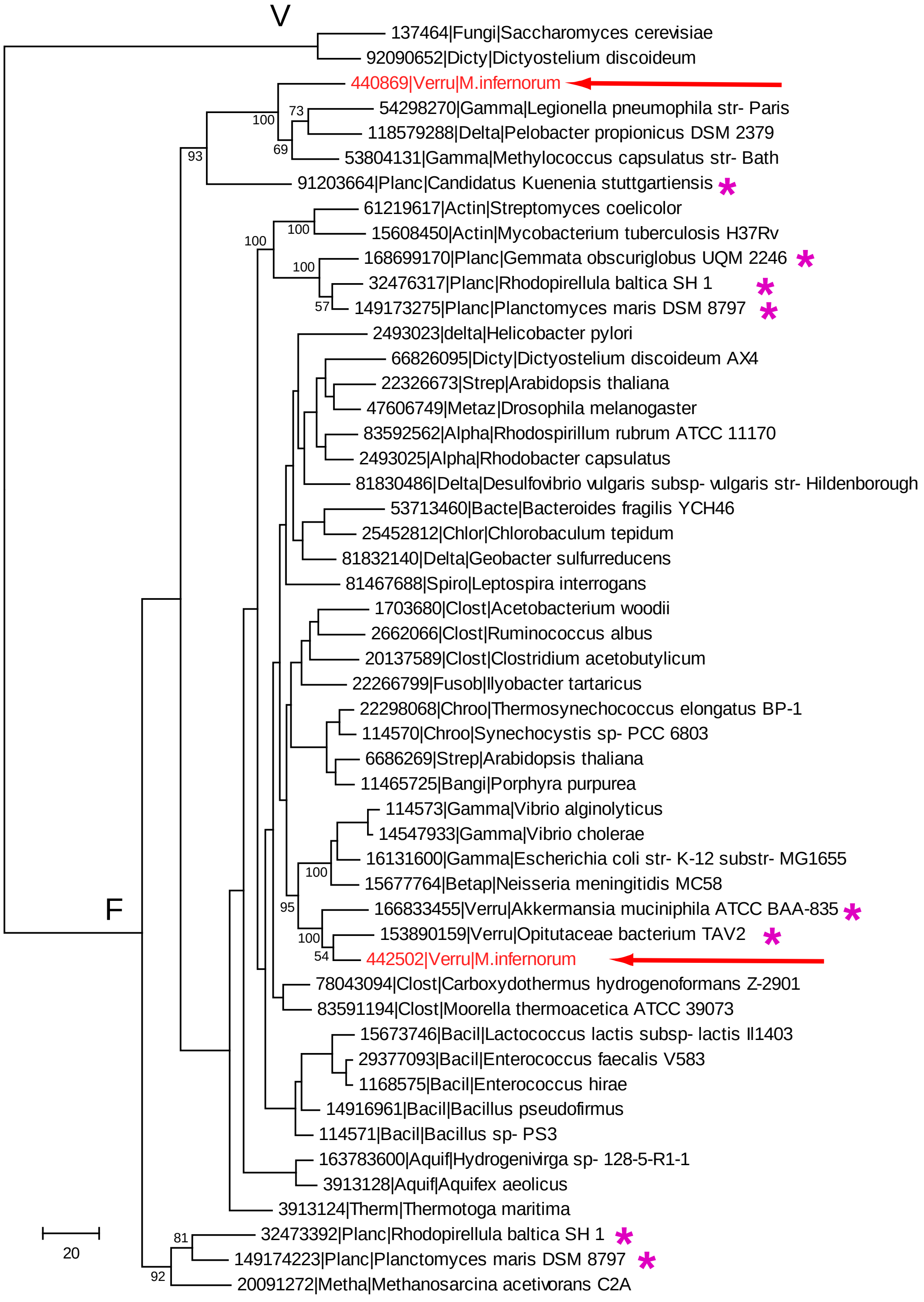

The tree was constructed as described in Methods and the legend to Supplementary Figure 1. A-subunits of V-ATPases from yeast and *D. discoideum* were used as an outgroup. Sequences from members of the PVC superphylum are indicated with asterisks; bootstrap values are shown for selected branches.

Supplementary Table 1. The list of 59 genomes, including "*Methylacidiphylum infernorum*"V4, used for genome comparisons and tree reconstruction in this work

Organism[a]	Taxonomy
Aquifex aeolicus VF5	*Aquificae*
Fervidobacterium nodosum Rt17-B1	*Thermotogae*
Thermotoga maritima MSB8	*Thermotogae*
Mycobacterium tuberculosis H37Rv	*Actinobacteria*
Bifidobacterium longum NCC2705	*Actinobacteria*
Rubrobacter xylanophilus DSM 9941	*Actinobacteria*
Synechocystis sp. PCC 6803	*Cyanobacteria*
Thermosynechococcus elongatus BP-1	*Cyanobacteria*
Gloeobacter violaceus PCC 7421	*Cyanobacteria*
Anabaena variabilis ATCC 29413	*Cyanobacteria*
Nostoc sp. PCC 7120	*Cyanobacteria*
Trichodesmium erythraeum IMS101	*Cyanobacteria*
Prochlorococcus marinus subsp. *marinus* str. CCMP1375	*Cyanobacteria*
Acaryochloris marina MBIC11017	*Cyanobacteria*
Chloroflexus aurantiacus J-10-fl	*Chloroflexi*
Dehalococcoides sp. BAV1	*Chloroflexi*
Deinococcus radiodurans R1	*Deinococcus/Thermus, Deinococcales*
Thermus thermophilus HB27	*Deinococcus/Thermus, Thermales*
Bacillus subtilis subsp. *subtilis* str. 168	*Firmicutes, Bacilli*
Lactobacillus casei ATCC 334	*Firmicutes, Bacilli*
Clostridium acetobutylicum ATCC 824	*Firmicutes, Clostridia*
Moorella thermoacetica ATCC 39073	*Firmicutes, Clostridia*
Mesoplasma florum L1	*Firmicutes, Mollicutes*
Fusobacterium nucleatum subsp. *nucleatum* ATCC 25586	*Fusobacteria*
Methylobacterium extorquens PA1	*Proteobacteria, Alphaproteobacteria*
Agrobacterium tumefaciens str. C58	*Proteobacteria, Alphaproteobacteria*
Rickettsia prowazekii str. Madrid E	*Proteobacteria, Alphaproteobacteria*
Burkholderia mallei ATCC 23344	*Proteobacteria, Betaproteobacteria*
Methylibium petroleiphilum PM1	*Proteobacteria, Betaproteobacteria*

Methylobacillus flagellatus KT	*Proteobacteria, Betaproteobacteria*
Neisseria meningitidis MC58	*Proteobacteria, Betaproteobacteria*
Escherichia coli K12	*Proteobacteria, Gammaproteobacteria*
Methylococcus capsulatus str. Bath	*Proteobacteria, Gammaproteobacteria*
Pseudomonas aeruginosa PAO1	*Proteobacteria, Gammaproteobacteria*
Desulfovibrio vulgaris subsp. *vulgaris* str. Hildenborough	*Proteobacteria, Deltaproteobacteria*
Myxococcus xanthus DK 1622	*Proteobacteria, Deltaproteobacteria*
Helicobacter pylori 26695	*Proteobacteria, Epsilonproteobacteria*
Sulfurovum sp. NBC37-1	*Proteobacteria, Epsilonproteobacteria*
Acidobacteria bacterium Ellin345	*Acidobacteria*
Solibacter usitatus Ellin6076	*Acidobacteria*
Bacteroides thetaiotaomicron VPI-5482	*Bacteroidetes/Chlorobi group, Bacteroidetes*
Flavobacterium johnsoniae UW101	*Bacteroidetes/Chlorobi group, Bacteroidetes*
Cytophaga hutchinsonii ATCC 33406	*Bacteroidetes/Chlorobi group, Bacteroidetes*
Chlorobium tepidum TLS	*Bacteroidetes/Chlorobi group, Chlorobi*
Prosthecochloris vibrioformis DSM 265	*Bacteroidetes/Chlorobi group, Chlorobi*
Leptospira interrogans serovar *Copenhageni* str. Fiocruz L1-130	*Spirochaetes*
Borrelia burgdorferi B31	*Spirochaetes*
Treponema pallidum subsp. *pallidum* str. Nichols	*Spirochaetes*
Gemmata obscuriglobus	*Planctomycetes*
Planctomyces maris DSM 8797	*Planctomycetes*
Rhodopirellula baltica SH 1	*Planctomycetes*
Blastopirellula marina DSM 3645	*Planctomycetes*
Chlamydia trachomatis D/UW-3/CX	*Chlamydiae/Verrucomicrobia group, Chlamydiae*
Chlamydophila pneumoniae AR39	*Chlamydiae/Verrucomicrobia group, Chlamydiae*
Candidatus *Protochlamydia amoebophila* UWE25	*Chlamydiae/Verrucomicrobia group, Chlamydiae*
Lentisphaera araneosa HTCC2155	*Chlamydiae/Verrucomicrobia group, Lentisphaerae*
Victivallis vadensis ATCC BAA-548	*Chlamydiae/Verrucomicrobia group, Lentisphaerae*
Opitutaceae bacterium TAV2	*Chlamydiae/Verrucomicrobia group, Verrucomicrobia*
Methylacidiphylum infernorum V4	*Chlamydiae/Verrucomicrobia group, Verrucomicrobia*

[a] The names of organisms with unfinished genome sequences are shaded with yellow.

Supplementary Table 2. Representation in "*Methylacidiphylum infernorum*" V4 of 12 protein families, selected by Strous *et al*. (2006) as "signature" proteins of the *Chlamydiae*/*Planctomycetes* superphylum[a]

No.	Orthologous group (OG)[a]	Orthologous group description	*Kuenenia* gene ID	Chlamydiae, Planctomycetes	Other bacteria	*Methylokorus infernorum*
1	NOG05013	60 kDa outer membrane protein	kustd1987	8, 2	0	no
2	COG04284	UDP-glucose pyrophosphorylase, involved in peptidoglycan, lipid A and LPS biosynthesis	kustc0526	8, 2	9	no
3	NOG43380	Polymorphic membrane protein precursor in *Chlamydiae*	kustd1438	7, 2	3	no
4	NOG06624	Lysine- and alanine-rich, basic proteins		8,1	5	no
5	COG01747	Contains a hypothetical N-terminal domain and a transcription elongation domain GreA	kustd1459	8,1	5	Minf_1252
6	COG03202	ATP/ADP translocase		8,1	9	no
7	COG03952	Lipid A-disaccharide synthase	kuste4276	8,1	10	Minf_2218
8	COG03880	Transmembrane protein with conserved CXXC motifs and a UVR domain, likely interacts with COG3869	kuste3071	8, 2	18	Minf_0201
9	COG03869	Member of the ATP:guanido phosphotransferase family, likely interacts with COG3880	kuste3072	8, 2	18	Minf_0242
10	COG02869	Na^+-translocating NADH dehydrogenase (ubiquinone) chain C	kuste3327	8, 2	19	no
11	COG01347	Na^+-translocating NADH dehydrogenase (ubiquinone) chain D	kustc0731	8, 2	19	no
12	COG02871	Na^+-translocating NADH-dehydrogenase (ubiquinone) chain F	kuste3329	8, 2	20	no

[a] Columns 2-6 are taken from the Supplementary Table 7 to the paper by Strous *et al*. (2006) Deciphering the evolution and metabolism of an anammox bacterium from a community genome, *Nature* **440**, 790-794. The 2nd column lists protein family codes in the STRING (http://string.embl.de) database. The 5th and 6th columns list the number of chlamydial (out of 8), planctomycetal (out of 2) and other bacterial (out of 157) genomes that encode members of the corresponding family.

Supplementary Table 3. Metabolic reconstruction of "*Methyloacidophylum infernorum*" V4

COG no.	Enzyme	EC no.	Gene	Pathway	Step	Minf locus
COG0686	Alanine dehydrogenase	EC 1.4.1.1	ald	Alanine biosynthesis	1	Minf_1758
COG0607	Rhodanese-related sulfurtransferase		pspE	Alanine biosynthesis	1	Minf_0494
COG5016	Pyruvate/oxaloacetate carboxyltransferase		-	Anaplerotic reaction providing intermediates of TCA		
COG2352	Phosphoenolpyruvate carboxylase	EC 4.1.1.31	Ppc	Anaplerotic reaction providing intermediates of TCA		Minf_1739
COG1038	Pyruvate carboxylase	EC 6.4.1.1	PycA	Anaplerotic reaction providing intermediates of TCA		
COG1246	N-acetylglutamate synthase		argA_2	Arginine biosynthesis	1	Minf_0290
COG0548	Acetylglutamate kinase	EC 2.3.1.1	argB	Arginine biosynthesis	2	Minf_0290
COG0002	Acetylglutamate semialdehyde dehydrogenase	EC 1.2.1.38	argC	Arginine biosynthesis	3	Minf_2363
COG4992	Ornithine/acetylornithine aminotransferase		-	Arginine biosynthesis	4	
COG0160	4-aminobutyrate aminotransferase	EC 2.6.1.11	argD	Arginine biosynthesis	4	Minf_1007
COG0624	Acetylornithine deacetylase/Succinyl-diaminopimelate desuccinylase	EC 3.5.1.6	argE	Arginine biosynthesis	5	Minf_0405
COG1364	N-acetylglutamate synthase	EC 2.3.1.1	argJ	Arginine biosynthesis	5	Minf_2362
COG0078	Ornithine carbamoyltransferase	EC 2.1.3.3	argF	Arginine biosynthesis	6	Minf_2359
COG0137	Argininosuccinate synthase	EC 6.3.4.5	argG	Arginine biosynthesis	7	Minf_1536
COG0165	Argininosuccinate lyase	EC 4.3.2.1	argH	Arginine biosynthesis	8	Minf_2428
COG0505	Carbamoylphosphate synthase small subunit	EC 6.3.5.5	carA	Arginine biosynthesis	9	Minf_1562
COG0458	Carbamoylphosphate synthase large subunit	EC 6.3.5.5	carB	Arginine biosynthesis	9	Minf_1932
COG2876	3-deoxy-D-arabino-heptulosonate 7-phosphate synthase	EC 2.5.1.54	aroA	Aromatic acids biosynthesis	1	
COG3200	3-deoxy-D-arabino-heptulosonate 7-phosphate synthase		aroG	Aromatic acids biosynthesis	1	
COG0722	3-deoxy-D-arabino-heptulosonate 7-phosphate synthase	EC 2.5.1.54	aroG	Aromatic acids biosynthesis	1	Minf_1352
COG0337	3-dehydroquinate synthetase	EC 4.2.3.4	aroB	Aromatic acids biosynthesis	2	Minf_0907
COG0710	3-dehydroquinate dehydratase	EC 4.2.1.10	aroD	Aromatic acids biosynthesis	3	Minf_1713
COG0757	dehydroquinate dehydratase II	EC 4.2.1.10	sll1112	Aromatic acids biosynthesis	3	

COG0169	Shikimate 5-dehydrogenase	EC 1.1.1.25	aroE	Aromatic acids biosynthesis	4	Minf_2478
COG0703	Shikimate kinase	EC 2.7.1.71	aroK	Aromatic acids biosynthesis	5	Minf_0185
COG1685	Archaeal shikimate kinase		MJ1440	Aromatic acids biosynthesis	5	
COG0128	5-enolpyruvylshikimate-3-phosphate synthase	EC 2.5.1.19	aroA	Aromatic acids biosynthesis	6	Minf_1047
COG0082	Chorismate synthase	EC 4.2.3.5	aroC	Aromatic acids biosynthesis	7	Minf_0184
COG1605	Chorismate mutase	EC 5.4.99.5	aroH, pheA_1	Aromatic acids biosynthesis	8	Minf_1711
COG0077	Prephenate dehydratase	EC 4.2.1.51	pheA_2	Aromatic acids biosynthesis	9	Minf_0961
COG0287	Prephenate dehydrogenase	EC 1.3.1.12	tyrA_2	Aromatic acids biosynthesis	9	Minf_0205
COG0079	Histidinol-phosphate/aromatic aminotransferase	EC 2.6.1.5	hisC	Aromatic acids biosynthesis	10	Minf_0206
COG1448	Aspartate/tyrosine/aromatic aminotransferase	EC 2.6.1.1	tyrB	Aromatic acids biosynthesis	10	
COG0436	Aspartate/tyrosine/aromatic aminotransferase	EC 2.6.1.1	-	Aspartate and Asparagine biosynthesis	1	Minf_0281
COG0029	Aspartate oxidase	EC 1.4.3.16	nadB	Aspartate and Asparagine biosynthesis	1	Minf_0927
COG2502	Asparagine synthetase A	EC 6.3.1.1	asnA	Aspartate and Asparagine biosynthesis	2	
COG0367	Asparagine synthase	EC 6.3.5.4	asnB	Aspartate and Asparagine biosynthesis	2	
COG1424	Pimeloyl-CoA synthetase	EC 6.2.1.14	BS_bioW	Biotin biosynthesis	1	
COG1042	Acyl-CoA synthetase		PA1017	Biotin biosynthesis	1	Minf_0021
COG0156	7-keto-8-aminopelargonate synthetase	EC 2.3.1.29	bioF	Biotin biosynthesis	2	Minf_1454
COG0161	Adenosylmethionine-8-amino-7-oxononanoate aminotransferase	EC 2.6.1.18	bioA	Biotin biosynthesis	3	Minf_1453
COG0132	Dethiobiotin synthetase	EC 6.3.3.3	bioD	Biotin biosynthesis	4	Minf_1148
COG0502	Biotin synthase	EC 2.8.1.6	bioB	Biotin biosynthesis	5	Minf_1134
COG0340	Biotin-(acetyl-CoA carboxylase) ligase	EC 6.3.4.15	birA_2	Biotin biosynthesis	6	Minf_1631
COG2038	NaMN:DMB phosphoribosyltransferase	EC 2.4.2.21	cobT	Cobalamine/B12 biosynthesis	1	
COG0007	Uroporphyrinogen-III methylase	EC 2.1.1.107	cysG_2/cobA	Cobalamine/B12 biosynthesis	2	Minf_2186
COG2243	Precorrin-2 methylase	EC 2.1.1.130	cbiL/cobI	Cobalamine/B12 biosynthesis	3	
COG0155	Sulfite reductase, beta subunit	EC 1.7.7.1	-/cobG	Cobalamine/B12 biosynthesis	4	Minf_1679
COG1010	Precorrin-3B methylase	EC 2.1.1.131	cbiH/cobJ	Cobalamine/B12 biosynthesis	5	
COG2875	Precorrin-4 methylase	EC 2.1.1.133	cbiF/cobM	Cobalamine/B12 biosynthesis	6	
COG2243	Precorrin-2 methylase	EC 2.1.1.130	-/cobF	Cobalamine/B12 biosynthesis	7	
COG2099	Precorrin-6x reductase	EC 1.3.1.54	cbiJ/cobK	Cobalamine/B12 biosynthesis	8	
COG2241	Precorrin-6B methylase 1	EC 2.1.1.132	cbiE/cobL_1	Cobalamine/B12 biosynthesis	9	
COG2242	Precorrin-6B methylase 2	EC 1.-.-.-	cbiT/cobL_2	Cobalamine/B12 biosynthesis	9	

COG2082	Precorrin isomerase	EC 5.4.1.2	cbiC/cobH	Cobalamine/B12 biosynthesis	10	
COG1797	Cobyrinic acid a,c-diamide synthase	EC 6.3.5.9	cbiA/cobB	Cobalamine/B12 biosynthesis	11	
COG1429	Cobalamin biosynthesis protein CobN	EC 6.6.1.2	-/cobN	Cobalamine/B12 biosynthesis	12	
COG2109	ATP:corrinoid adenosyltransferase	EC 2.5.1.17	cobA/cobO	Cobalamine/B12 biosynthesis	13	
COG1492	Cobyric acid synthase	EC 6.3.5.10	cbiP/cobQ	Cobalamine/B12 biosynthesis	14	
COG1270	Cobalamin biosynthesis protein CobD/CbiB	EC 6.3.1.10	cbiB/cobD	Cobalamine/B12 biosynthesis	15	
COG2087	Adenosyl cobinamide kinase/adenosyl cobinamide phosphate guanylyltransferase	EC 2.7.1.156	cobU	Cobalamine/B12 biosynthesis	16	
COG2266	GTP:adenosylcobinamide-phosphate guanylyltransferase		cobY	Cobalamine/B12 biosynthesis	16	
COG0368	Cobalamin-5-phosphate synthase	EC 2.7.8.26	cobS	Cobalamine/B12 biosynthesis	17	
COG1045	Serine acetyltransferase	EC 2.3.1.30	cysE	Cysteine biosynthesis	1	Minf_2477
COG0110	Acetyltransferase		WbbJ	Cysteine biosynthesis	1	
COG0031	Cysteine synthase	EC 2.5.1.47	cysK,cysM	Cysteine biosynthesis	2	Minf_0228
COG0403	Glycine cleavage system protein P, N-terminal domain	EC 1.4.4.2	gcvP_1	Degradation of glycine	1	
COG1003	Glycine cleavage system protein P, C-terminal domain	EC 1.4.4.2	gcvP_2	Degradation of glycine	1	Minf_2170
COG1249	Dihydrolipoamide dehydrogenase	EC 1.6.1.1	lpd	Degradation of glycine	2	Minf_0069
COG0509	Glycine cleavage system H protein		gcvH	Degradation of glycine	2	Minf_2168
COG0404	Glycine cleavage system T protein	EC 2.1.2.10	gcvT	Degradation of glycine	2	Minf_2167
COG0364	Glucose-6-phosphate 1-dehydrogenase	EC 1.1.1.49	zwf	Entner-Doudoroff pathway	1	Minf_0262
COG0363	6-phosphogluconolactonase/Glucosamine-6-phosphate isomerase/deaminase	EC 3.1.1.31	nagB	Entner-Doudoroff pathway	2	Minf_1495
COG0129	Phosphogluconate dehydratase	EC 4.2.1.9	ilvD	Entner-Doudoroff pathway	3	Minf_2260
COG0800	2-keto-3-deoxy-6-phosphogluconate aldolase	EC 4.1.2.14	eda	Entner-Doudoroff pathway	4	
COG0825	Acetyl-CoA carboxylase alpha subunit	EC 6.4.1.2	accA	Fatty acids biosynthesis	1	Minf_1665
COG0511	Biotin carboxyl carrier protein	EC 4.1.1.41	accB	Fatty acids biosynthesis	1	Minf_1640
COG0439	Biotin carboxylase	EC 6.3.4.14	accC	Fatty acids biosynthesis	1	Minf_0366
COG0777	Acetyl-CoA carboxylase beta subunit	EC 6.4.1.2	accD	Fatty acids biosynthesis	1	Minf_1668
COG0331	(acyl-carrier-protein) S-malonyltransferase	EC 2.3.1.39	fabD	Fatty acids biosynthesis	2	Minf_0433
COG0332	3-oxoacyl-[acyl-carrier-protein] synthase III	EC 2.3.1.41	fabH	Fatty acids biosynthesis	3	Minf_1216
COG1028	Dehydrogenases with different	EC 1.1.1.30	fabG	Fatty acids biosynthesis	4	Minf_0432

	specificities					
COG0764	3-hydroxymyristoyl/3-hydroxydecanoyl-(acyl carrier protein) dehydratases	EC 4.2.1.60	fabA	Fatty acids biosynthesis	5	Minf_2131
COG0623	Enoyl-[acyl-carrier-protein] reductase	EC 1.3.1.9	fabI	Fatty acids biosynthesis	6	Minf_1633
COG0304	3-oxoacyl-(acyl-carrier-protein) synthase	EC 2.3.1.41	fabB	Fatty acids biosynthesis	7	Minf_0430
COG0183	Acetyl-CoA acetyltransferase	EC 2.3.1.9	paaJ	Fatty acids metabolism	1	
COG1250	3-hydroxyacyl-CoA dehydrogenase	EC 1.1.1.35	fadB	Fatty acids metabolism	2	
COG1024	Enoyl-CoA hydratase/carnithine racemase	EC 4.2.1.17	caiD	Fatty acids metabolism	3	
COG1960	Acyl-CoA dehydrogenases	EC 1.3.99.-	caiA	Fatty acids metabolism	4	
COG0236	Acyl carrier protein		acpP	Fatty acids metabolism	5	Minf_0431
COG0302	GTP cyclohydrolase I	EC 3.5.4.16	folE	Folate biosynthesis	1	
COG1469	GTP cyclohydrolase I			Folate biosynthesis	1	
COG1785	Alkaline phosphatase	EC 3.1.3.1	phoA	Folate biosynthesis	2	
COG1539	Dihydroneopterin aldolase	EC 4.1.2.25	folB	Folate biosynthesis	3	Minf_1823
COG0801	7,8-dihydro-6-hydroxymethylpterin-pyrophosphokinase	EC 2.7.6.3	folK	Folate biosynthesis	4	Minf_0340
COG0294	Dihydropteroate synthase	EC 2.5.1.15	folP	Folate biosynthesis	5	Minf_1898
COG0285	Folylpolyglutamate synthase	EC 6.3.2.17	folC	Folate biosynthesis	6	Minf_0385
COG0262	Dihydrofolate reductase	EC 1.5.1.3	folA	Folate biosynthesis	7	
COG1866	Phosphoenolpyruvate carboxykinase	EC 4.1.1.49	pckA	Gluconeogenesis		Minf_0887
COG0281	Malic enzyme. NAD or NADPH dependent	EC 1.1.1.38	sfcA	Gluconeogenesis		
COG0158	Fructose-1,6-bisphosphatase	EC 3.1.3.11	fbp	Gluconeogenesis only		Minf_1684
COG0574	Phosphoenolpyruvate synthase/pyruvate phosphate dikinase	EC 2.7.9.1	ppsA	Gluconeogenesis only		Minf_0020
COG0334	Glutamate dehydrogenase/leucine dehydrogenase	EC 1.4.1.2	gdhA	Glutamate and Glutamine biosynthesis	1	Minf_1762
COG0174	Glutamine synthetase	EC 6.3.1.2	glnA	Glutamate and Glutamine biosynthesis	2	Minf_1203
COG0067	Glutamate synthase domain 1	EC 1.4.7.1	gltB_1	Glutamate and Glutamine biosynthesis	3	Minf_2281
COG0069	Glutamate synthase domain 2	EC :1.4.1.13	gltB_2	Glutamate and Glutamine biosynthesis	3	Minf_2281
COG0070	Glutamate synthase domain 3	EC :1.4.1.13	gltB_3	Glutamate and Glutamine biosynthesis	3	Minf_2281
COG0493	NADPH-dependent glutamate synthase beta chain	EC 1.4.1.13	gltD	Glutamate and Glutamine biosynthesis	4	Minf_0627
COG0554	Glycerol kinase		glpK	Glycerol metabolism		Minf_1703

COG1929	Glycerate kinase	EC 2.7.1.31	glxK	Glycerol metabolism		
COG0837	Glucokinase	EC 2.7.1.2	glk	Glycolysis	1	Minf_2003
COG0205	6-phosphofructokinase	EC 2.7.1.90	pfkA	Glycolysis	3	Minf_2122
COG0524	Sugar kinases, ribokinase family	EC 2.7.1.4	pfkB	Glycolysis	3	Minf_1083
COG0469	Pyruvate kinase	EC 2.7.1.40	pykK	Glycolysis	9	Minf_0082
COG0166	Glucose-6-phosphate isomerase	EC 5.3.1.9	pgi	Glycolysis/Gluconeogenesis	2	Minf_1017
COG0191	Fructose/tagatose bisphosphate aldolase	EC 4.1.2.13	fba	Glycolysis/Gluconeogenesis	4	Minf_0287
COG0149	Triosephosphate isomerase	EC 5.3.1.1	tpi	Glycolysis/Gluconeogenesis	4	Minf_1031
COG0057	Glyceraldehyde-3-phosphate dehydrogenase	EC 1.2.1.12	gapA	Glycolysis/Gluconeogenesis	5	Minf_1033
COG0126	3-phosphoglycerate kinase	EC 2.7.2.3	pgk	Glycolysis/Gluconeogenesis	6	Minf_1032
COG0588	Phosphoglycerate mutase 1	E5.4.2.1	gpmA	Glycolysis/Gluconeogenesis	7	Minf_0600
COG0406	Fructose-2,6-bisphosphatase	EC 2.7.1.105	gpmB	Glycolysis/Gluconeogenesis	7	Minf_1259
COG0696	Phosphoglyceromutase	E5.4.2.1	yibO	Glycolysis/Gluconeogenesis	7	
COG0148	Enolase	EC 4.2.1.11	eno	Glycolysis/Gluconeogenesis	8	Minf_0312
COG2224	Isocitrate lyase	EC 4.1.3.1	aceA	Glyoxylate bypass	1	Minf_0891
COG2225	Malate synthase	EC 2.3.3.9	aceB	Glyoxylate bypass	2	Minf_0892
COG0008	Glutamyl- and glutaminyl-tRNA synthetases	EC 6.1.1.17	gltX	Heme biosynthesis	1	Minf_0178
COG0373	Glutamyl-tRNA reductase	EC 1.2.1.-	hemA	Heme biosynthesis	2	Minf_2188
COG0001	Glutamate-1-semialdehyde aminotransferase	EC 5.4.3.8	hemL	Heme biosynthesis	3	Minf_1903
COG0113	Delta-aminolevulinic acid dehydratase	EC 4.2.1.24	hemB	Heme biosynthesis	4	Minf_1112
COG0181	Porphobilinogen deaminase	EC 2.5.1.61	hemC	Heme biosynthesis	5	Minf_2187
COG1587	Uroporphyrinogen-III synthase	EC 4.2.1.75	hemD	Heme biosynthesis	6	Minf_2186
COG1648	Siroheme synthase	EC 1.3.1.76	cysG_1	Heme biosynthesis	7	
COG0007	Uroporphyrinogen-III methylase	EC 2.1.1.107	cysG_2	Heme biosynthesis	7	Minf_2186
COG0407	Uroporphyrinogen-III decarboxylase	EC 4.1.1.37	hemE	Heme biosynthesis	7	
COG0408	Coproporphyrinogen III oxidase	EC 1.3.3.3	hemF	Heme biosynthesis	8	
COG0635	Coproporphyrinogen III oxidase	EC 1.3.3.3	hemN	Heme biosynthesis	8	Minf_0310
COG0716	Flavodoxins	EC 1.3.3.4	hemG	Heme biosynthesis	9	
COG1232	Protoporphyrinogen oxidase	EC 1.3.3.4	hemY	Heme biosynthesis	9	
COG0276	Protoheme ferro-lyase	EC 4.99.1.1	hemH	Heme biosynthesis	10	
COG0462	Phosphoribosylpyrophosphate synthetase	EC 2.7.6.1	prsA	Histidine biosynthesis	1	Minf_0666
COG0040	Ammonia permease	EC 2.4.2.17	hisG	Histidine biosynthesis	2	Minf_0818
COG0140	Phosphoribosyl-ATP pyrophosphohydrolase	EC 3.6.1.31	hisI_2	Histidine biosynthesis	3	
COG0139	Phosphoribosyl-AMP cyclohydrolase	EC 3.5.4.19	hisI_1	Histidine biosynthesis	4	Minf_0056
COG0106	Phosphoribosylformimino-5-	EC 5.3.1.16	hisA	Histidine biosynthesis	5	Minf_1927

	aminoimidazole carboxamide ribonucleotide isomerase					
COG0107	Imidazoleglycerol-phosphate synthase		hisF	Histidine biosynthesis	6	Minf_2270
COG0118	Glutamine amidotransferase	EC 2.4.2.-	hisH	Histidine biosynthesis	6	Minf_1926
COG0131	Imidazoleglycerol-phosphate dehydratase	EC 4.2.1.19	hisB_2	Histidine biosynthesis	7	Minf_2371
COG0079	Histidinol-phosphate/aromatic aminotransferase and cobyric acid decarboxylase	EC 2.6.1.5	hisC	Histidine biosynthesis	8	Minf_0206
COG0241	Histidinol phosphatase	EC 3.1.3.15	Bs_ytvB	Histidine biosynthesis	9	Minf_0958
COG1387	Histidinol phosphatase	EC 3.1.3.15	hisB_1	Histidine biosynthesis	9	Minf_0443
COG0141	Histidinol dehydrogenase	EC 1.1.1.23	hisD	Histidine biosynthesis	10	Minf_0243
COG1171	Threonine dehydratase	EC 4.3.1.19	ilvA	Ile, Leu, Val biosynthesis	1	
COG0028	Aacetolactate synthase	EC 1.2.2.2	ilvB	Ile, Leu, Val biosynthesis	2	Minf_2367
COG0440	Acetolactate synthase, small subunit	EC 2.2.1.6	ilvN	Ile, Leu, Val biosynthesis	2	Minf_1745
COG0059	Ketol-acid reductoisomerase	EC 1.1.1.86	ilvG	Ile, Leu, Val biosynthesis	3	Minf_1744
COG0129	Dihydroxyacid dehydratase/phosphogluconate dehydratase	EC 4.2.1.9	ilvD	Ile, Leu, Val biosynthesis	4	Minf_2260
COG0436	Aspartate/tyrosine/aromatic aminotransferase	EC 2.6.1.1	avtA	Ile, Leu, Val biosynthesis	5	Minf_0281
COG0115	Branched-chain amino acid aminotransferase	EC 2.6.1.21	ilvE	Ile, Leu, Val biosynthesis	5	Minf_0202
COG0119	Isopropylmalate/homocitrate/citramalate synthases	EC 4.1.3.4	leuA	Ile, Leu, Val biosynthesis	6	Minf_1743
COG0065	3_isopropylmalate dehydratase large subunit	EC 4.2.1.33	leuC	Ile, Leu, Val biosynthesis	7	Minf_0745
COG0066	3_isopropylmalate dehydratase small subunit	EC 4.2.1.33	leuD	Ile, Leu, Val biosynthesis	7	Minf_1565
COG0473	Isocitrate/isopropylmalate dehydrogenase	EC 1.1.1.41	leuB	Ile, Leu, Val biosynthesis	8	Minf_0939
COG0115	Branched-chain amino acid aminotransferase	EC 2.6.1.21	ilvE	Ile, Leu, Val biosynthesis	9	Minf_0202
COG1448	Aspartate/tyrosine/aromatic aminotransferase	EC 2.6.1.1	tyrB	Ile, Leu, Val biosynthesis	9	
COG0578	Glycerol-3-phosphate dehydrogenase	EC 1.1.1.8	gpsA	Isoprenoid (branch) mevalonate biosynthesis	1	
COG0240	Glycerol-3-phosphate dehydrogenase	EC 1.1.1.8	gpsA	Isoprenoid (branch) mevalonate biosynthesis	1	Minf_0182
COG2376	Dihydroxyacetone kinase	EC 2.7.1.29		Isoprenoid (branch) mevalonate biosynthesis	1	

COG0371	Glycerol dehydrogenase	EC 1.1.1.6	GldA	Isoprenoid (branch) mevalonate biosynthesis	2	
COG1646	GGGPS,-3-O-geranylgeranylglyceryl phosphate synthase		-	Isoprenoid (branch) mevalonate biosynthesis	3	
COG1304	L-lactate dehydrogenase	EC 1.1.2.3	- (lldD)	Isoprenoid deoxyxylylose pathway	1	
COG1154	Deoxyxylulose-5-phosphate synthase	EC 2.2.1.7	dxs	Isoprenoid deoxyxylylose pathway	1	Minf_1537
COG0743	1-deoxy-D-xylulose 5-phosphate reductoisomerase	EC 1.1.1.267	dxr (yaeM)	Isoprenoid deoxyxylylose pathway	2	Minf_1972
COG0142	Geranylgeranyl pyrophosphate synthase	EC 2.5.1.1	ispA, ispB	Isoprenoid deoxyxylylose pathway	2	Minf_0397
COG1211	4-diphosphocytidyl-2-methyl-D-erithritol synthase	EC 2.7.7.60	ispD (ygbP)	Isoprenoid deoxyxylylose pathway	3	Minf_0787
COG1947	4-diphosphocytidyl-2C-methyl-D-erythritol 2-phosphate synthase	EC 2.7.1.148	ispE(ychB)	Isoprenoid deoxyxylylose pathway	4	Minf_1286
COG0245	2C-methyl-D-erythritol 2,4-cyclodiphosphate synthase	EC 4.6.1.12	ispF(ygbB)	Isoprenoid deoxyxylylose pathway	5	Minf_0771
COG0821	4-hydroxy-3-methylbut-2-en-1-yl diphosphate synthase	EC 1.17.4.3	gcpE	Isoprenoid deoxyxylylose pathway	6	Minf_1968
COG0761	4-hydroxy-3-methylbut-2-enyl diphosphate reductase	EC 1.17.1.2	lytB	Isoprenoid deoxyxylylose pathway	7	Minf_2119
COG3425	3-hydroxy-3-methylglutaryl CoA synthase	EC 2.3.3.10	pksG	Isoprenoid mevalonate biosynthesis	1	
COG1257	Hydroxymethylglutaryl-CoA reductase	EC 1.1.1.34	HMG1	Isoprenoid mevalonate biosynthesis	2	
COG1577	Mevalonate kinase	EC 2.7.1.36	ERG12	Isoprenoid mevalonate biosynthesis	3	
COG3890	Phosphomevalonate kinase		ERG8	Isoprenoid mevalonate biosynthesis	4	
COG3407	Mevalonate pyrophosphate decarboxylase	EC 4.1.1.33	-	Isoprenoid mevalonate biosynthesis	5	
COG1443	Isopentenyldiphosphate isomerase	EC 5.3.3.2	Idi	Isoprenoid mevalonate biosynthesis	6	
COG0142	Geranylgeranyl pyrophosphate synthase	EC 2.5.1.1	ispA, ispB	Isoprenoid mevalonate biosynthesis	7	Minf_0397
COG1043	Acyl-[acyl carrier protein]--UDP-N-acetylglucosamine O-acyltransferase	EC 2.3.1.129	lpxA	Lipid A biosynthesis	1	Minf_0223
COG0774	UDP-3-O-acyl-N-acetylglucosamine deacetylase	EC 3.5.1.-	lpxC	Lipid A biosynthesis	2	Minf_2131
COG1044	UDP-3-O-[3-hydroxymyristoyl] glucosamine N-acyltransferase	EC 2.3.1.-	lpxD	Lipid A biosynthesis	3	Minf_0748

COG0763	Lipid A disaccharide synthetase	EC 2.4.1.182	lpxB	Lipid A biosynthesis	4	Minf_1859
COG1663	Tetraacyldisaccharide-1-P 4'-kinase	EC 2.7.1.130	lpxK	Lipid A biosynthesis	5	Minf_1888
COG2877	3-deoxy-D-manno-octulosonic acid 8-phosphate synthase	EC 2.5.1.55	kdsA	Lipid A biosynthesis	6	Minf_0954
COG1212	CMP-2-keto-3-deoxyoctulosonic acid synthetase	EC 2.7.7.38	kdsB	Lipid A biosynthesis	7	Minf_0956
COG1519	3-deoxy-D-manno-octulosonic-acid transferase	EC 2.-.-.-	kdtA	Lipid A biosynthesis	8	Minf_1736
COG1560	Lauroyl/myristoyl acyltransferase	EC 2.3.1.-	htrB, msbB	Lipid A biosynthesis	9	
COG0320	Lipoate synthase		lipA	Lipoic acid biosynthesis	1	Minf_1525
COG0321	Lipoate-protein ligase B		lipB	Lipoic acid/protein (protein lipoylation) biosynthesis	2	
COG0095	Lipoate-protein ligase A		lplA	Lipoic acid/protein (protein lipoylation) biosynthesis	2	Minf_1759
COG0119	Isopropylmalate/homocitrate/citramalate synthases	EC 4.1.3.4	lysS	Lysine biosynthesis – alpha aminoadipate pathway	1	Minf_1743
COG0065	3-isopropylmalate dehydratase large subunit	EC 4.2.1.33	lysT	Lysine biosynthesis – alpha aminoadipate pathway	2	Minf_0745
COG0066	3-isopropylmalate dehydratase small subunit	EC 4.2.1.33	lysU	Lysine biosynthesis – alpha aminoadipate pathway	2	Minf_1565
COG0189	Glutathione synthase	EC 6.3.2.3	lysX	Lysine biosynthesis – alpha aminoadipate pathway	5	
COG0548	Acetylglutamate kinase	EC 2.3.1.1	lysZ	Lysine biosynthesis – alpha aminoadipate pathway	6	Minf_0290
COG0002	Acetylglutamate semialdehyde dehydrogenase	EC 1.2.1.38	lysY/ArdC1	Lysine biosynthesis – alpha aminoadipate pathway	7	Minf_2363
COG4992	Ornithine/acetylornithine aminotransferase		argD, lysJ	Lysine biosynthesis – alpha aminoadipate pathway	8	Minf_0947
COG0624	Acetylornithine deacetylase/ Succi nyl-diaminopimelate desuccinylase	EC 3.5.1.6	argE	Lysine biosynthesis – alpha aminoadipate pathway	9	Minf_0405
	Function unclear	#N/A	lysW	Lysine biosynthesis – alpha aminoadipate pathway	10	
COG0527	Aspartokinases	EC 2.7.2.4	lysC	Lysine biosynthesis – diaminopimelate pathway	1	Minf_2090
COG0136	Aspartate-semialdehyde dehydrogenase	EC 1.2.1.11	asd	Lysine biosynthesis – diaminopimelate pathway	2	Minf_1460
COG0329	Dihydrodipicolinate synthase/N-acetylneuraminate lyase	EC 4.1.3.3	dapA	Lysine biosynthesis – diaminopimelate pathway	3	Minf_1924
COG0289	Dihydrodipicolinate reductase	EC 1.3.1.26	dapB	Lysine biosynthesis –	4	Minf_1923

				diaminopimelate pathway		
COG2171	Tetrahydrodipicolinate N-succinyltransferase	EC 2.3.1.117	dapD	Lysine biosynthesis – diaminopimelate pathway	5	
COG		#N/A	dapC	Lysine biosynthesis – diaminopimelate pathway	6	
COG0624	Acetylornithine deacetylase/Succinyl-diaminopimelate desuccinylase	EC 3.5.1.6	dapE	Lysine biosynthesis – diaminopimelate pathway	7	Minf_0405
COG0253	Diaminopimelate epimerase	EC 5.1.1.7	dapF	Lysine biosynthesis – diaminopimelate pathway	8	Minf_1925
COG0019	Diaminopimelate decarboxylase	EC 4.1.1.20	lysA	Lysine biosynthesis – diaminopimelate pathway	9	Minf_1078
COG0527	Aspartokinases	EC 2.7.2.4	metL_1	Methionine biosynthesis	1	Minf_2090
COG0136	Aspartate-semialdehyde dehydrogenase	EC 1.2.1.11	asd	Methionine biosynthesis	2	Minf_1460
COG0460	Homoserine dehydrogenase	EC 1.1.1.3	metL_2	Methionine biosynthesis	3	Minf_0747
COG1897	Homoserine trans-succinylase	EC 2.3.1.46	metA	Methionine biosynthesis	4	
COG0626	Cystathionine beta-lyase/ cystathionine gamma-synthases	EC 2.5.1.48	metB	Methionine biosynthesis	5	
COG0626	Cystathionine beta-lyase/ cystathionine gamma-synthases	EC 2.5.1.48	metC	Methionine biosynthesis	6	
COG2021	Homoserine acetyltransferase	EC 2.3.1.31	met2	Methionine biosynthesis	7	
COG2873	O-acetylhomoserine sulfhydrylase	EC 2.5.1.49	met17	Methionine biosynthesis	8	
COG0620	Methionine synthase II	EC 2.1.1.14	metE	Methionine biosynthesis	9	Minf_0258
COG0646	Methionine synthase I, methyltransferase domain	EC 2.1.1.13	metH_1	Methionine biosynthesis	9	Minf_1622
COG1410	Methionine synthase I, cobalamin-binding domain	EC 2.1.1.13	metH_2	Methionine biosynthesis	9	
COG0192	S-adenosylmethionine synthetase	EC 2.5.1.6	metK	Methionine biosynthesis	10	Minf_0670
COG2896	Molybdenum cofactor biosynthesis enzyme		moaA	Mo Factor biosynthesis	1	Minf_1883
COG0521	Molybdopterin biosynthesis enzymes		moaB	Molibdopterin biosynthesis	1	Minf_0053
COG0315	Molybdenum cofactor biosynthesis enzyme		moaC	Molibdopterin biosynthesis	1	Minf_0052
COG1977	Molybdopterin converting factor, small subunit		moaD	Molibdopterin biosynthesis	2	Minf_1249
COG0314	Molybdopterin converting factor, large subunit		moaE	Molibdopterin biosynthesis	2	Minf_1881
COG0746	Molybdopterin-guanine dinucleotide biosynthesis protein A		mobA	Molibdopterin biosynthesis	3	Minf_0971
COG1763	Molybdopterin-guanine dinucleotide		mobB	Molibdopterin biosynthesis	3	Minf_0972

	biosynthesis protein					
COG0476	Molybdopterin and thiamine biosynthesis enzyme		moeB	Molibdopterin biosynthesis	3	Minf_0654
COG0725	ABC-type molybdate transport system, periplasmic component		modA	Molibdopterin biosynthesis	4	Minf_0987
COG4149	ABC-type molybdate transport system, permease component		modB	Molibdopterin biosynthesis	4	Minf_0988
COG4148	ABC-type molybdate transport system, ATPase component		modC	Molibdopterin biosynthesis	4	
COG0157	Nicotinate-nucleotide pyrophosphorylase	EC 2.4.2.19	modD	Molibdopterin biosynthesis	5	Minf_2412
COG0303	Molybdopterin biosynthesis enzyme		moeA	Molibdopterin biosynthesis	5	Minf_0973
COG0521	Molybdopterin biosynthesis enzymes		mog	Molibdopterin biosynthesis	5	Minf_0053
COG0766	UDP-N-acetylglucosamine enolpyruvyl transferase	EC 2.5.1.7	murA	Mureine biosynthesis	1	Minf_0426
COG0812	UDP-N-acetylmuramate dehydrogenase	EC 1.1.1.158	murB	Mureine biosynthesis	2	Minf_1409
COG0773	UDP-N-acetylmuramate-alanine ligase	EC 6.3.2.8	murC	Mureine biosynthesis	3	Minf_1409
COG0771	UDP-N-acetylmuramoylalanine-D-glutamate ligase	EC 6.3.2.9	murD	Mureine biosynthesis	4	Minf_2246
COG0769	UDP-N-acetylmuramyl tripeptide synthase	EC 6.3.2.13	murE	Mureine biosynthesis	5	Minf_1415
COG0770	UDP-N-acetylmuramyl pentapeptide synthase	EC 6.3.2.10	murF	Mureine biosynthesis	6	Minf_1414
COG0472	UDP-N-acetylmuramyl pentapeptide phosphotransferase	EC 2.7.8.13	mraY	Mureine biosynthesis	7	Minf_1413
COG0707	UDP-N-acetylglucosamine:LPS N-acetylglucosamine transferase	EC 2.4.1.227	murG	Mureine biosynthesis	8	Minf_1632
COG0796	Glutamate racemase	EC 5.1.1.3	murI	Mureine biosynthesis	9	
COG1181	D-alanine-D-alanine ligase	EC 6.3.2.4	ddlA(B)	Mureine biosynthesis	10	Minf_1408
COG0787	Alanine racemase	EC 5.1.1.1	air	Mureine biosynthesis	11	Minf_0070
COG0029	Aspartate oxidase	EC 1.4.3.16	nadB	NAD biosynthesis	1	Minf_0927
COG0379	Quinolinate synthase		nadA	NAD biosynthesis	2	Minf_0006
COG0157	Nicotinate-nucleotide pyrophosphorylase	EC 2.4.2.19	nadC	NAD biosynthesis	3	Minf_2412
COG1488	Nicotinic acid phosphoribosyltransferase	EC 2.4.2.11	pncB	NAD biosynthesis	4	Minf_0838
COG1057	Nicotinic acid mononucleotide adenylyltransferase	EC 2.7.7.18	nadD	NAD biosynthesis	5	Minf_1361
COG0171	NAD synthase	EC 6.3.5.1	nadE	NAD biosynthesis	6	Minf_0725
COG1056	Nicotinamide mononucl	EC 2.7.7.1	nadR_2	NAD biosynthesis	5	
COG0028	acetolactate synthase	EC 1.2.2.2	ilvB	Pantothenate and CoA biosynthesis	1	Minf_2367
COG0059	Ketol-acid reductoisomerase	EC 1.1.1.86	ilvC	Pantothenate and CoA biosynthesis	2	Minf_1744

COG0129	Dihydroxyacid dehydratase/phosphogluconate dehydratase	EC 4.2.1.9	ilvD	Pantothenate and CoA biosynthesis	3	
COG0413	Ketopantoate hydroxymethyltransferase	EC 2.1.2.11	PanB	Pantothenate and CoA biosynthesis	4	Minf_0341
COG1893	Ketopantoate reductase	EC 1.1.1.169	PanE/apbA	Pantothenate and CoA biosynthesis	5	Minf_0239
COG0414	Panthothenate synthetase	EC 6.3.2.1	PanC	Pantothenate and CoA biosynthesis	6	Minf_2101
COG1072	Panthothenate kinase	EC 2.7.1.33	CoaA	Pantothenate and CoA biosynthesis	7	
COG0452	Phosphopantothenoylcysteine synthetase/decarboxylase		dfp	Pantothenate and CoA biosynthesis	8	Minf_1524
COG0669	Phosphopantetheine adenylyltransferase	EC 2.7.7.3	CoaD	Pantothenate and CoA biosynthesis	9	Minf_0425
COG0237	Dephospho-CoA kinase	EC 2.7.1.24	CoaE	Pantothenate and CoA biosynthesis	10	Minf_0445
COG0364	Glucose-6-phosphate 1-dehydrogenase	EC 1.1.1.49	zwf	Pentose phosphate pathway	1	Minf_0262
COG0363	6-phosphogluconolactonase	EC 3.1.1.31	nagB	Pentose phosphate pathway	2	Minf_1495
COG0362	6-phosphogluconate dehydrogenase	EC 1.1.1.44	gnd	Pentose phosphate pathway	3	Minf_0198
COG0120	Ribose 5-phosphate isomerase	EC 5.3.1.6	rpiA	Pentose phosphate pathway	4	
COG0698	Ribose 5-phosphate isomerase	EC 5.3.1.6	rpiB	Pentose phosphate pathway	4	Minf_0197
COG0021	Transketolase	EC 2.2.1.1	tktA	Pentose phosphate pathway	5	Minf_1260
COG0176	Transaldolase	EC 2.2.1.2	mipB	Pentose phosphate pathway	6	Minf_1118
COG0036	Pentose-5-phosphate-3-epimerase	EC 5.1.3.1	rpe	Pentose phosphate pathway	7	Minf_0606
COG0235	Ribulose-5-phosphate 4-epimerase	EC 4.1.2.17	araD	Pentose phosphate pathway	8	
COG2937	Glycerol-3-phosphate O-acyltransferase	EC 2.3.1.15	PlsB	Phospholipid biosynthesis	1	
COG0204	1-acyl-sn-glycerol-3-phosphate acyltransferase	EC 2.3.1.51	PlsC	Phospholipid biosynthesis	2	Minf_1048
COG0575	CDP-diglyceride synthetase	EC 2.7.7.41	CdsA	Phospholipid biosynthesis	3	Minf_0014
COG1502	Phosphatidylserine/ phosphatidyl-glycerophosphate/ cardiolipin synthases	EC 2.7.8.8	Cls	Phospholipid biosynthesis	4	
COG0688	Phosphatidylserine decarboxylase	EC 4.1.1.65	Psd	Phospholipid biosynthesis	5	Minf_0015
COG0558	Phosphatidylglycerophosphate synthase	EC 2.7.8.5	PgsA	Phospholipid biosynthesis	4'	Minf_0177
COG1267	Phosphatidylglycerophosphatase A	EC 3.1.3.27	PgpA	Phospholipid biosynthesis	5'	
COG1502	Phosphatidylserine/ phosphatidyl-glycerophosphate/ cardiolipin synthases	EC 2.7.8.8	Cls	Phospholipid biosynthesis	6'	
COG0671	Membrane-associated phospholipid phosphatase	EC 3.1.3.27	PgpB	Phospholipid biosynthesis	7'	Minf_1207
COG1246	N-acetylglutamate synthase		argA_2	Proline biosynthesis	1	Minf_0290
COG1364	N-acetylglutamate synthase	EC 2.3.1.1	BS_argJ	Proline biosynthesis	1	Minf_2362
COG0548	Acetylglutamate kinase	EC 2.3.1.1	argB	Proline biosynthesis	2	Minf_0290
COG0002	Acetylglutamate semialdehyde dehydrogenase	EC 1.2.1.38	argC	Proline biosynthesis	3	Minf_2363
COG0160	4-aminobutyrate aminotransferase	EC 2.6.1.11	argD	Proline biosynthesis	4	Minf_1007

COG0624	Acetylornithine deacetylase/ Succinyl-diaminopimelate desuccinylase	EC 3.5.1.6	argE	Proline biosynthesis	5	Minf_0405
COG2423	Predicted ornithine cyclodeaminase	EC 4.3.1.12	AF1665	Proline biosynthesis	6	
COG0345	Pyrroline-5-carboxylate reductase	EC 1.5.1.2	proC	Proline biosynthesis	7	Minf_0186
COG0506	Proline dehydrogenase	EC 1.5.99.8	putA_1	Proline biosynthesis	8	Minf_0717
COG0014	Gamma-glutamyl phosphate reductase	EC 1.2.1.41	proA	Proline biosynthesis	9	Minf_0809
COG1012	NAD-dependent aldehyde dehydrogenases	EC 1.2.1.3	putA_2	Proline biosynthesis	10	Minf_1730
COG0263	Glutamate 5-kinase	EC 2.7.2.11	proB	Proline biosynthesis	11	Minf_0808
COG0788	Formyltetrahydrofolate hydrolase	EC 3.5.1.10	PurU	Purine salvage	1	
COG0813	Purine-nucleoside phosphorylase	EC 2.4.2.1	DeoD	Purine salvage	2	
COG0005	Purine nucleoside phosphorylase	EC 2.4.2.1	Pnp	Purine salvage	2	
COG0503	Adenine/guanine phosphoribosyltransferases	EC 2.4.2.7	Apt	Purine salvage	3	Minf_0226
COG0634	Hypoxanthine-guanine phosphoribosyltransferase	EC 2.4.2.8	Hpt	Purine salvage	4	Minf_1659
COG1052	Lactate dehydrogenase	EC 1.1.1.29	pdxB	Pyridoxal phosphate/B6 biosynthesis	1	Minf_1323
COG1154	Deoxyxylulose-5-phosphate synthase	EC 2.2.1.7	dxs	Pyridoxal phosphate/B6 biosynthesis	2	Minf_1537
COG1995	Pyridoxal phosphate biosynthesis protein	EC 1.1.1.262	pdxA	Pyridoxal phosphate/B6 biosynthesis	3	Minf_0291
COG0854	Pyridoxal phosphate biosynthesis protein		pdxJ	Pyridoxal phosphate/B6 biosynthesis	3	Minf_0766
COG2240	Pyridoxal/pyridoxine/ pyridoxamine kinase	EC 2.7.1.35	pdxK	Pyridoxal phosphate/B6 biosynthesis	4	
COG0259	Pyridoxamine-phosphate oxidase	EC 1.4.3.5	pdxH	Pyridoxal phosphate/B6 biosynthesis	5	Minf_1150
		#N/A		Pyridoxal phosphate/B6 biosynthesis	1	
COG1932	Phosphoserine aminotransferase	EC 2.6.1.52	serC	Pyridoxal phosphate/B6 biosynthesis		
COG0214	Pyridoxine biosynthesis enzyme		SNZ1 (BS_yaaD)	Pyridoxal phosphate/B6 biosynthesis	1	
COG0311	Glutamine amidotransferase involved in pyridoxine biosynthesis		SNZ2	Pyridoxal phosphate/B6 biosynthesis	2	
COG0505	Carbamoylphosphate synthase small subunit	EC 6.3.5.5	carA	Pyrimidine biosynthesis	1	Minf_1562
COG0458	Carbamoylphosphate synthase large	EC 6.3.5.5	carB	Pyrimidine biosynthesis	1	Minf_1932

	subunit					
COG0540	Aspartate carbamoyltransferase, catalytic chain	EC 2.1.3.2	pyrB	Pyrimidine biosynthesis	2	Minf_1564
COG0418	Dihydroorotase	E3.5.2.3	pyrC	Pyrimidine biosynthesis	3	
COG0044	Dihydroorotase	EC 3.5.2.2	ydeZ	Pyrimidine biosynthesis	3	Minf_0625
COG0167	Dihydroorotate dehydrogenase	EC 1.3.3.1	pyrD	Pyrimidine biosynthesis	4	Minf_0626
COG0461	Orotate phosphoribosyltransferase	EC 2.4.2.10	pyrE	Pyrimidine biosynthesis	5	Minf_1893
COG0284	Orotidine-5'-phosphate decarboxylase	EC 4.1.1.23	pyrF	Pyrimidine biosynthesis	6	Minf_1285
COG0563	Adenylate kinase	EC 2.7.4.3	adk	Pyrimidine biosynthesis	7	Minf_0822
COG0528	Uridylate kinase		pyrH	Pyrimidine biosynthesis	7	Minf_1813
COG0105	Nucleoside diphosphate kinase	EC 2.7.4.6	ndk	Pyrimidine biosynthesis	8	Minf_1367
COG0504	CTP synthase	EC 6.3.4.2	pyrG	Pyrimidine biosynthesis	9	Minf_0955
COG0295	Cytidine deaminase	EC 3.5.4.5	Cdd	Pyrimidine salvage	1	
COG0402	Cytosine deaminase	EC 3.5.4.1	SsnA	Pyrimidine salvage	2	Minf_1455
COG0035	Uracil phosphoribosyltransferase	EC 2.4.2.9	Upp	Pyrimidine salvage	3	Minf_1111
COG0572	Uridine kinase	EC 2.7.1.19	Udk	Pyrimidine salvage	4	Minf_1261
COG0813	Purine-nucleoside phosphorylase	EC 2.4.2.1	DeoD	Pyrimidine salvage	5	
COG0213	Thymidine phosphorylase	EC 2.4.2.2	DeoA	Pyrimidine salvage	6	
COG0208	Ribonucleotide reductase beta subunit	EC 1.17.4.1	NrdF	Pyrimidine salvage	7	Minf_1077
COG0209	Ribonucleotide reductase alpha subunit	EC 1.17.4.1	NrdA	Pyrimidine salvage	8	Minf_1076
COG0775	Nucleoside phosphorylase	EC 3.2.2.4	Pfs	Pyrimidine salvage	9	Minf_0400
COG1435	Thymidine kinase	EC 2.7.1.21	Tdk	Pyrimidine salvage	10	
COG0462	Phosphoribosylpyrophosphate synthetase	EC 2.7.6.1	prsA	Pyrine biosynthesis	1	Minf_0666
COG0034	Glutamine phosphoribosylpyrophosphate amidotransferase	EC 2.4.2.14	purF	Pyrine biosynthesis	2	Minf_1218
COG0151	Phosphoribosylamine-glycine ligase	EC 6.3.4.13	purD	Pyrine biosynthesis	3	Minf_1728
COG0299	Folate-dependent phosphoribosyl glycinamide formyltransferase PurN	EC 2.1.2.2	purN	Pyrine biosynthesis	4	Minf_0795
COG0027	Formate-dependent phosphoribosyl glycinamide formyltransferase		purT	Pyrine biosynthesis	4	
COG0046	Phosphoribosylformylglycinamidine synthase, synthetase domain	EC 6.3.5.3	purL_1	Pyrine biosynthesis	5	Minf_0721
COG0047	Phosphoribosylformylglycinamidine synthase, glutamine amidotransferase domain	EC 6.3.5.3	purL_2	Pyrine biosynthesis	5	Minf_2304
COG0150	Phosphoribosylaminoimidazole synthetase	EC 6.3.3.1	purM	Pyrine biosynthesis	6	Minf_1219
COG0026	Phosphoribosylaminoimidazole carboxylase	EC 4.1.1.21	purK	Pyrine biosynthesis	7	Minf_2026

COG0041	Phosphoribosylcarboxyaminoimidazole mutase	EC 4.1.1.21	purE	Pyrine biosynthesis	8	Minf_2027
COG0152	Phosphoribosylaminoimidazolesuccinocarboxamide synthase	EC 6.3.2.6	purC	Pyrine biosynthesis	9	Minf_1401
COG0015	Adenylosuccinate lyase	EC 4.3.2.2	purB	Pyrine biosynthesis	10	Minf_2302
COG0138	AICAR transformylase/IMP cyclohydrolase PurH	EC 2.1.2.3	purH	Pyrine biosynthesis	11	Minf_1126
COG0104	Adenylosuccinate synthase	EC 6.3.4.4	purA	Pyrine biosynthesis	12	Minf_0011
COG0516	IMP dehydrogenase/GMP reductase	EC 1.7.1.7	guaB, guaC	Pyrine biosynthesis	13	Minf_0245
COG0015	Adenylosuccinate lyase	EC 4.3.2.2	purB	Pyrine biosynthesis	14	Minf_2302
COG0518	GMP synthase - Glutamine amidotransferase domain	EC 6.3.5.2	guaA_1	Pyrine biosynthesis	15	Minf_0244
COG0519	GMP synthase, PP-ATPase domain/subunit	EC 6.3.5.2	guaA_2	Pyrine biosynthesis	15	Minf_0244
COG2609	Pyruvate dehydrogenase complex, dehydrogenase component	EC 1.2.4.1	AceE	Pyruvate oxidation	1(1)	
COG0508	Pyruvate/2-oxoglutarate dehydrogenase complex, dihydrolipoamide acyltransferase component	EC 2.3.1.12	AceF	Pyruvate oxidation	1(1)	Minf_0068
COG1071	Pyruvate/2-oxoglutarate dehydrogenase complex, dehydrogenase component	EC 1.2.4.1	AcoA	Pyruvate oxidation	1(1)	Minf_1006
COG1249	Pyruvate/2-oxoglutarate dehydrogenase complex, dihydrolipoamide dehydrogenase component	EC 1.6.1.1	Lpd	Pyruvate oxidation	1(1)	Minf_0069
COG1144	Pyruvate:ferredoxin oxidoreductase	EC 1.2.7.1	-	Pyruvate oxidation	1(2)	Minf_0626
COG0674	Pyruvate:ferredoxin oxidoreductase	EC 1.2.7.1	porA	Pyruvate oxidation	1(2)	
COG1013	Pyruvate:ferredoxin oxidoreductase	EC 1.2.7.1	porB	Pyruvate oxidation	1(2)	Minf_2386
COG1014	Pyruvate:ferredoxin oxidoreductase	EC 1.2.7.1	porG	Pyruvate oxidation	1(2)	Minf_1239
COG1180	Pyruvate-formate lyase-activating enzyme		pflA	Pyruvate oxidation	1(3)	Minf_0640
COG1882	Pyruvate-formate lyase	EC 2.3.1.54	pflD	Pyruvate oxidation	1(3)	
COG1313	Pyruvate formate lyase activating protein		pflX	Pyruvate oxidation	1(3)	
COG3961	Pyruvate decarboxylase		-	Pyruvate oxidation	1(4)	Minf_2197
COG0807	GTP cyclohydrolase II	EC 3.5.4.25	ribA	Riboflavin biosynthesis	1	Minf_2430
COG0117	Pyrimidine deaminase	EC 3.5.4.26	ribD_1	Riboflavin biosynthesis	2	Minf_1660
COG1985	Pyrimidine reductase, riboflavin biosynthesis	EC 1.1.1.193	ribD_2	Riboflavin biosynthesis	2	Minf_1660
COG0108	3,4-dihydroxy-2-butanone 4-phosphate synthase		ribB	Riboflavin biosynthesis	3	Minf_2430

COG1731	Archaeal riboflavin synthase		MJ1184	Riboflavin biosynthesis	4	
COG0307	Riboflavin synthase alpha chain	EC 2.5.1.9	ribC	Riboflavin biosynthesis	4	Minf_2397
COG0054	Riboflavin synthase beta-chain	EC 2.5.1.9	ribE	Riboflavin biosynthesis	4	Minf_1664
COG0111	Phosphoglycerate dehydrogenase	EC 1.1.1.95	serA	Serine, glycine biosynthesis	1	Minf_1255
COG0075	Serine-pyruvate aminotransferase	EC 2.6.1.-	agt1	Serine, glycine biosynthesis	2	Minf_0603
COG1932	Phosphoserine aminotransferase	EC 2.6.1.52	serC	Serine, glycine biosynthesis	2	
COG4359	Methylthioadenosine recycling enzyme		BS_ykrX	Serine, glycine biosynthesis	3	
COG0560	Phosphoserine phosphatase	EC 3.1.3.3	serB	Serine, glycine biosynthesis	3	
COG0112	Glycine/serine hydroxymethyltransferase	EC 2.1.2.1	glyA	Serine, glycine biosynthesis	4	Minf_1669
COG0175	3'-phosphoadenosine 5'-phosphosulfate sulfotransferase	EC 1.8.4.8	cysD	Sulfate reduction	1	Minf_1878
COG2895	GTPases - Sulfate adenylate transferase	EC 2.7.7.4	cysN	Sulfate reduction	1	Minf_1880
COG2046	ATP sulfurylase	EC 2.7.7.4	met3	Sulfate reduction	1	
COG0529	Adenylylsulfate kinase	EC 2.7.1.25	cysC	Sulfate reduction	2	Minf_1880
COG0175	3'-phosphoadenosine 5'-phosphosulfate sulfotransferase	EC 1.8.4.8	cysH	Sulfate reduction	3	Minf_1878
COG0155	Sulfite reductase, beta subunit	EC 1.7.7.1	cysI	Sulfate reduction	4	Minf_1679
COG0369	Sulfite reductase, alpha subunit	EC 1.8.1.2	cysJ	Sulfate reduction	4	Minf_0216
COG0372	Citrate synthase	EC 2.3.3.1	gltA	TCA cycle	1	Minf_0334
COG1048	Aconitase A	EC 4.2.1.3	acnA	TCA cycle	2	Minf_1027
COG1049	Aconitase B	EC 4.2.1.3	acnB	TCA cycle	2	
COG0538	Isocitrate dehydrogenases	EC 1.1.1.42	icd	TCA cycle	3	
COG0473	Isocitrate/isopropylmalate dehydrogenase	EC 1.1.1.41	IDH2	TCA cycle	3	Minf_0939
COG0567	2-oxoglutarate dehydrogenase complex, dehydrogenase component,	EC 1.2.4.2	sucA	TCA cycle	4	Minf_0067
COG0508	Dihydrolipoamide acyltransferase,	EC 2.3.1.12	sucB	TCA cycle	4	Minf_0068
COG1249	Dihydrolipoamide dehydrogenase	EC 1.6.1.1	sucD	TCA cycle	4	Minf_0069
COG0045	Succinyl-CoA synthetase, beta subunit	EC 6.2.1.4	sdhA	TCA cycle	5	Minf_1045
COG0074	Succinyl-CoA synthetase, alpha subunit	EC 6.2.1.4	sdhB	TCA cycle	5	Minf_1046
COG1053	Succinate dehydrogenase/fumarate reductase, flavoprotein subunit	EC 1.3.5.1	fumA_1	TCA cycle	6	Minf_0388
COG0479	Succinate dehydrogenase/fumarate reductase, Fe-S protein subunit	EC 1.3.5.1	fumA_2	TCA cycle	6	Minf_0389
COG0114	Fumarase	EC 4.2.1.2	fumC	TCA cycle	7	Minf_0793
COG1951	Tartrate dehydratase alpha subunit/Fumarate hydratase class I, N-terminal domain	EC 4.2.1.2	lpd	TCA cycle	7	
COG1838	Tartrate dehydratase beta subunit/Fumarate hydratase class I, C-	EC 4.2.1.2	sucC	TCA cycle	7	

	terminal domain					
COG0039	Malate/lactate dehydrogenases	EC 1.1.1.27	mdh	TCA cycle	8	Minf_1373
COG2055	Malate/L-lactate dehydrogenases	EC 1.1.1.37	ybiC	TCA cycle	8	
COG1154	Deoxyxylulose-5-phosphate synthase	EC 2.2.1.7	dxs	Thiamine (B1) biosynthesis	1	Minf_1537
COG0422	Thiamine biosynthesis protein ThiC		ThiC	Thiamine (B1) biosynthesis	1	Minf_0066
COG0351	Hydroxymethylpyrimidine/phosphomethylpyrimidine kinase	EC 2.7.4.7	ThiD	Thiamine (B1) biosynthesis	2	Minf_1574
COG2104	Sulfur transfer protein involved in thiamine biosynthesis		ThiS	Thiamine (B1) biosynthesis	2	Minf_2025
COG0352	Thiamine monophosphate synthase	EC 2.5.1.3	ThiE	Thiamine (B1) biosynthesis	3	Minf_0367
COG0476	Dinucleotide-utilizing enzymes involved in molybdopterin and thiamine biosynthesis family 2		ThiF	Thiamine (B1) biosynthesis	3	Minf_0654
COG0301	Thiamine biosynthesis ATP pyrophosphatase		ThiI	Thiamine (B1) biosynthesis	3	
COG2022	Uncharacterized enzyme of thiazol biosynthesis		ThiG	Thiamine (B1) biosynthesis	4	Minf_2404
COG1060	Thiamine biosynthesis enzyme ThiH		ThiH	Thiamine (B1) biosynthesis	4	Minf_1916
COG0611	Thiamine monophosphate kinase	EC 2.7.4.16	ThiL	Thiamine (B1) biosynthesis	4	Minf_0073
COG0352	Thiamine monophosphate synthase	EC 2.5.1.3	ThiE	Thiamine (B1) biosynthesis	5	Minf_0367
COG2145	Hydroxyethylthiazole kinase, sugar kinase family	EC 2.7.1.50	ThiM(=ThiK)	Thiamine (B1) biosynthesis	5	
COG1564	Thiamine pyrophosphokinase	EC 2.7.6.2	TH180	Thiamine (B1) biosynthesis	6	
COG0611	Thiamine monophosphate kinase	EC 2.7.4.16	ThiL	Thiamine (B1) biosynthesis	6	Minf_0073
COG0527	Aspartokinases	EC 2.7.2.4	thrA_1	Threonine biosynthesis	1	Minf_2090
COG0136	Aspartate-semialdehyde dehydrogenase	EC 1.2.1.11	asd	Threonine biosynthesis	2	Minf_1460
COG0460	Homoserine dehydrogenase	EC 1.1.1.3	thrA_2	Threonine biosynthesis	3	Minf_0747
COG0083	Homoserine kinase	EC 2.7.1.39	thrB	Threonine biosynthesis	4	Minf_2410
COG0498	Threonine synthase	EC 4.2.3.1	thrC	Threonine biosynthesis	5	Minf_0746
COG1328	Oxygen-sensitive ribonucleoside-triphosphate reductase	EC 1.17.4.2	nrdD	Thymidylate biosynthesis	1	Minf_1076
COG0717	Deoxycytidine deaminase	EC 3.5.4.13	dcd	Thymidylate biosynthesis	2	Minf_0086
COG0209	Ribonucleotide reductase alpha subunit	EC 1.17.4.1	nrdA	Thymidylate biosynthesis	3	Minf_1076
COG0208	Ribonucleotide reductase beta subunit	EC 1.17.4.1	nrdF	Thymidylate biosynthesis	4	Minf_1077
COG0105	Nucleoside diphosphate kinase	EC 2.7.4.6	ndk	Thymidylate biosynthesis	5	Minf_1367
COG0756	dUTPase	EC 3.6.1.23	dut	Thymidylate biosynthesis	6	
COG4508	Dimeric dUTPase			Thymidylate biosynthesis	6	Minf_2265
COG1351	Predicted alternative thymidylate synthase	EC 2.1.1.148	THY1	Thymidylate biosynthesis	7	

COG0207	Thymidylate synthase	EC 2.1.1.45	thyA	Thymidylate biosynthesis	7	Minf_0110
COG1531	Uncharacterized ArCR		-	Thymidylate biosynthesis	8	
COG0125	Thymidylate kinase	EC 2.7.4.9	tmk	Thymidylate biosynthesis	9	Minf_1637
COG0105	Nucleoside diphosphate kinase	EC 2.7.4.6	ndk	Thymidylate biosynthesis	10	Minf_1367
COG2876	3-deoxy-D-arabino-heptulosonate 7-phosphate synthase	EC 2.5.1.54	aroA	Tryptophan biosynthesis	1	
COG3200	3-deoxy-D-arabino-heptulosonate 7-phosphate synthase		aroG	Tryptophan biosynthesis	1	
COG0722	3-deoxy-D-arabino-heptulosonate 7-phosphate synthase	EC 2.5.1.54	aroG	Tryptophan biosynthesis	1	Minf_1352
COG0337	3-dehydroquinate synthetase	EC 4.2.3.4	aroB	Tryptophan biosynthesis	2	Minf_0907
COG0710	3-dehydroquinate dehydratase	EC 4.2.1.10	aroD	Tryptophan biosynthesis	3	Minf_1713
COG0757	3-dehydroquinate dehydratase II	EC 4.2.1.10	sll1112(aroQ)	Tryptophan biosynthesis	3	
COG0169	Shikimate 5-dehydrogenase	EC 1.1.1.25	aroE	Tryptophan biosynthesis	4	Minf_2478
COG0703	Shikimate kinase	EC 2.7.1.71	aroK	Tryptophan biosynthesis	5	Minf_0185
COG1685	Archaeal shikimate kinase		MJ1440	Tryptophan biosynthesis	5	
COG0128	5-enolpyruvylshikimate-3-phosphate synthase	EC 2.5.1.19	aroA	Tryptophan biosynthesis	6	Minf_1047
COG0082	Chorismate synthase	EC 4.2.3.5	aroC	Tryptophan biosynthesis	7	Minf_0184
COG0512	Anthranilate/para-aminobenzoate synthases component II	EC 4.1.3.27	trpD_1	Tryptophan biosynthesis	8	Minf_1763
COG0147	Anthranilate/para-aminobenzoate synthases component I	EC 4.1.3.27	trpE	Tryptophan biosynthesis	8	Minf_1764
COG0547	Anthranilate phosphoribosyltransferase	EC 2.4.2.18	trpD_2	Tryptophan biosynthesis	9	Minf_1561
COG0135	Phosphoribosylanthranilate isomerase	EC 5.3.1.24	trpC_2	Tryptophan biosynthesis	10	Minf_2031
COG0134	Indole-3-glycerol phosphate synthase	EC 4.1.1.48	trpC_1	Tryptophan biosynthesis	11	Minf_2032
COG0159	Tryptophan synthase alpha chain	EC 4.2.1.20	trpA	Tryptophan biosynthesis	12	Minf_2278
COG0133	Tryptophan synthase beta chain	EC 4.2.1.20	trpB	Tryptophan biosynthesis	12	Minf_2030
COG1350	Predicted alternative tryptophan synthase beta-subunit		trpB2	Tryptophan biosynthesis	12	
COG2876	3-deoxy-D-arabino-heptulosonate 7-phosphate synthase	EC 2.5.1.54	aroA	Ubiquinol biosynthesis	1	
COG3200	3-deoxy-D-arabino-heptulosonate 7-phosphate synthase		aroG	Ubiquinol biosynthesis	1	
COG0722	3-deoxy-D-arabino-heptulosonate 7-phosphate synthase	EC 2.5.1.54	aroG	Ubiquinol biosynthesis	1	Minf_1352
COG0337	3-dehydroquinate synthetase	EC 4.2.3.4	aroB	Ubiquinol biosynthesis	2	Minf_0907
COG0710	3-dehydroquinate dehydratase	EC 4.2.1.10	aroD	Ubiquinol biosynthesis	3	Minf_1713
COG0757	3-dehydroquinate dehydratase II	EC 4.2.1.10	aroQ (sll1112)	Ubiquinol biosynthesis	3	

COG0169	Shikimate 5-dehydrogenase	EC 1.1.1.25	aroE	Ubiquinol biosynthesis	4	Minf_2478
COG0703	Shikimate kinase	EC 2.7.1.71	aroK	Ubiquinol biosynthesis	5	Minf_0185
COG1685	Archaeal shikimate kinase		MJ1440	Ubiquinol biosynthesis	5	
COG0128	5-enolpyruvylshikimate-3-phosphate synthase	EC 2.5.1.19	aroA	Ubiquinol biosynthesis	6	Minf_1047
COG0082	Chorismate synthase	EC 4.2.3.5	aroC	Ubiquinol biosynthesis	7	Minf_0184
COG1169	Isochorismate synthase	EC 5.4.4.2	menF	Ubiquinol biosynthesis	8	
COG1165	2-succinyl-6-hydroxy-2,4-cyclohexadiene-1-carboxylate synthase	EC 4.1.1.71	menD	Ubiquinol biosynthesis	9	
COG1441	O-succinylbenzoate synthase	EC 4.2.1.6	menC	Ubiquinol biosynthesis	10	
COC0318	Acyl-CoA synthetases/AMP-acid ligases II	EC 6.2.1.26	menE	Ubiquinol biosynthesis	11	
COG0447	Dihydroxynaphthoic acid synthase	EC 4.1.3.36	menB	Ubiquinol biosynthesis	12	
COG1575	1,4-dihydroxy-2-naphthoate octaprenyltransferase	EC 2.5.1.-	menA	Ubiquinol biosynthesis	13	
COG0684	Demethylmenaquinone methyltransferase	EC 2.1.1.-	menG	Ubiquinol biosynthesis	14	
COG0505	Carbamoylphosphate synthase small subunit	EC 6.3.5.5	carA	Urea cycle	1	Minf_1562
COG0458	Carbamoylphosphate synthase large subunit	EC 6.3.5.5	carB	Urea cycle	1	Minf_1932
COG0078	Ornithine carbamoyltransferase	EC 2.1.3.3	argF	Urea cycle	2	Minf_2359
COG0137	Argininosuccinate synthase	EC 6.3.4.5	argG	Urea cycle	3	Minf_1536
COG0165	Argininosuccinate lyase	EC 4.3.2.1	argH	Urea cycle	4	Minf_2428
COG0010	Arginase/agmatinase/ formimionoglutamate hydrolase	EC 3.5.3.1	speB	Urea cycle	5	
