# Supplementary material for: Complete genome sequence of the extremely acidophilic methanotroph isolate V4, Methylacidiphilum infernorum, a representative of the bacterial phylum Verrucomicrobia
Source: Biol Direct. 2008 Jul 1;3:26. doi: 10.1186/1745-6150-3-26 (PMC2474590; doi:10.1186/1745-6150-3-26)
Supplement: Additional file 2 — Supplementary Figures added in the course of the Open Review. Supplementary Figures 7–9 [file 1745-6150-3-26-S2.pdf]

Supplementary Figure 7. Taxonomic affiliations of “*Methylacidiphilum infernorum*” V4 proteins.

Taxonomic affiliations of the best BLAST hits for each of the *M. infernorum* proteins to the RefSeq database before (top panel, identical to Figure 3) and after (bottom panel) the inclusion in RefSeq of the proteins encoded in four members of *Verrucomicrobia: Akkermansia muciniphila*, bacterium Ellin514, *Opitutus terrae*, and *Verrucomicrobium spinosum.*

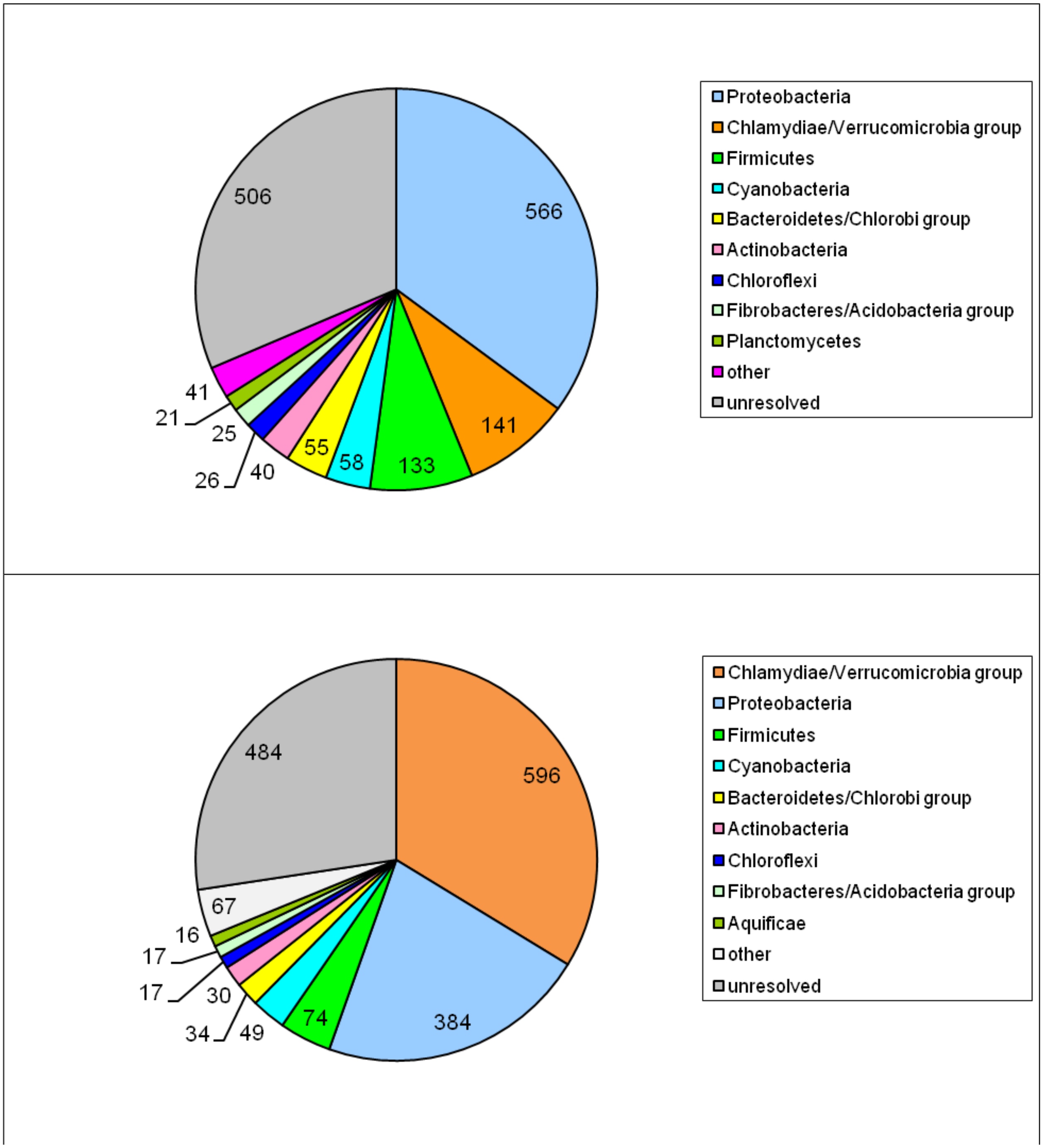

Supplementary Figure 8 (created by Prof. R. S. Gupta). Multiple alignment of the second protein family specific for the Planctomyces/Verrucomicrobia/ Chlamydia group.

```
Chlamydia trachomatis D/UW-3/CX# NP_219933      MSRHRSYGKSV-KGETKRNVLKRFERIEVLRKLGRWDDATAKKATGLLKTPAIK
Chlamydia muridarum Nigg NP_297078              MSRHRSYGKSI-KGETKRNVLKRFERIEVLRKLGRWDDATAKKATGLLKTPVIK
Chlamydophila felis Fe/C-56 YP_515687           MSRHRSYGKSI-KGETKRNVLKRFERIEVLRKLGRWNDATAKKATGLPKTPVMK
Chlamydophila caviae GPIC NP_829109             MSRHRSYGKSI-KGETKRNVLKRFERIDVLRKLGRWNDVTAKKATGLPKTPVMK
Chlamydophila abortus S26/3 YP_219659           MSRHRSYGKSI-KGETKRNVLKRFERIDLLRKLSRWNDTTAKKATGLPKTPVIK
Chlamydia pneumoniae CWL029# NP_224704          MSRHRSYGKSV-KGVTKRNVLKRFERVEVLRKLGRWNDSTAKKVTGLPKTPILK
Protochlamydia amoebophila UWE25 YP_008052      MSRHPSFGKAG-KTATKRNVLKRFERIDVLKKIGRWKDSENKRITGLPKTPVL-
Bacterium Ellin 514 ZP_02965781                 MSQHRSLRAVA-TMGGKRNVLKRFERVGLLKKRGQWKE--GDRITGLRKTKPEA
Lentisphaera araneosa HTCC 2155 ZP_01877577     MSIHRSLKVKG-NTAGKKNVLKRFERVDQLIQEGRLKP--GDQVLGLPKTKVNI
Akkermansia muciniphila BAA-835 YP_001876867    MSKHSSLKATG-TVGGKRSVLKRFERVKLLKERGEWKK--GQSPLGLPKTKHEA
Verrucomicrobium spinosum DSM 4136 ZP_02926670  MSQHRSLKSSGGSVGTKRSVLKRGERIKLMKARGQWNE--GRSLYNLPKTKPEA
Opitutus terrae PB90-1 YP_001819597             MSQHKSLQGSS-GIVIKRNVLKRFERVDILKKRGQWKA--GDRVQGLRKTKPDV
Planctomyces maris DSM 8797 ZP_01856939         VSLDKSLKSKS-TLVRARNVLKRAERIEKLKFEDRWVE--GQGALGLPKVRVEK
C. Kuenenia stuttgartiensis CAJ71823            MSIDKSLKPKG-KLSRPRNVYRKVERIAILKAENRWDQ--DTSVFGLPKVKVEK
M. infernorum V4 Minf_0061 YP_001938720.2##     MSQHRSYRTGS-LLVAKRNVLKRYERINILKKQGKWKE--GDKVLGLPKTKPI-
                                                :*...*.... ......:.* :: **:......  ..... .....* *.
```

Multiple sequence alignment of the protein CT421.2 (NP_219933), for which all BLAST hits are observed from the PVC group (*Planctomycetes*, *Verrucomicrobia*, *Lentisphaerae* and *Chlamydiae*) species. The full-length sequences are shown for most species (length 51-53 aa), except for the two *Planctomycetes*, where sequences are 81 aa long and contain extra amino acids at the N – or C-terminal ends. The positions that are identical in all sequences are marked with *. Other conserved positions are marked with the symbols : and .

[#] In *C. trachomatis* and *C. pneumoniae*, the protein is present in all of the sequenced strains and the sequences are identical to those shown here.

[##]The updated sequence of Minf_0061 (YP_001938720.2) was added by the authors.

Supplementary Figure 9 (created by Prof. R. S. Gupta). Partial sequence alignment of the RpoB protein.

			176		224
Proteobacteria	E. coli	CAA23629	IIPYRGSWLDFEFDPKDNLFVRID		RRRKLPATIILRALNYTTEQ ILDLF
	V. cholerae	EAY40360	-------------------Y----		-------S------GK-SAE ---I-
	Pse. aeruginosa	NP_252960	-----------------CV-----		-------SV-----G-S--E --NA-
	Pas. multocida	NP_246676	-------------------YA---		-------------------- ---I-
	Ral. solanacearum	NP_521155	-----------------I-YF-V-		----M-V--L-KSIGL-P-- --AH-
	Nei. meningitidis	YP_974254	-----------------L-YF---		----M-V--L-K--G-NN-- ---I-
	A. tumefaciens	NP_354931	V---------I---A--IVYA---		------V-SL-M--GMDG-E --ST-
	Ca. crescentus	NP_419321	V-------------A--IVY----		--------TF-Y--GMDG-E --TT-
	Geo. sulfurreducens	NP_953905	V-------------H--I------		----M---VL-K--G-SA-A L-NY-
	Bde. bacteriovorus	NP_969761	V---------A---Q--LIH----		----F-V--L-K--G-NS-- L-EY-
	Hel. pylori	NP_207989	---D-----Y--Y-S--V-YA--N		K---V-V--LF--MD-QKQD -IKM-
	Camp. jejuni	YP_178546	---D-----Y--Y-A--V-Y---N		K---V-V-MLF---G-KKQD -IK--
Aquificales	Hydrogenobaculum Y04AAS1	ZP_02061359	-H-DK--RIE--LSTNSDIISARI		DKKRIS-IS----FGLE-AYD--SQ-
	Aqu. aeolicus	NP_214331	---DK--RVE--LSGATDLISARI		D-K--S--AV---FGLE-AYD--KY-
Chlamydiae	Chl. muridarum	NP_296965	---------EAS--IN-LIYIH--	RKK	----IL-MTFI---G-S-DAD-IEE-
	Chl. trachomatis	NP_219820	---------EAV--IN-LIYIH--	RKK	----IL-MTFI---G-S-DAD-IEE-
	Chlam. pneumoniae	NP_445236	---------EAI--IN-LIYIH--	RKK	----IL-ITFI---G-SSDAD-IEE-
	Chlam. abortus	YP_220059	---------EAI--IN-LIYIH--	RKK	----IL-ITFI---G-SSDAD-IEE-
	Chlam. felis	YP_515237	---------EAI--IN-LIYIH--	RKK	----IL-MTFI---G-SSDAD-IEE-
	Chlam. caviae	NP_829555	---------EAI--IN-LIYIH--	RKK	----IL-MTFI---G-SSDAD-IEE-
	Chlam. psittaci	AAV92911	---------EAI--IN-LIYIH--	RKK	----IL-ITFI---G-SSDAD-IEE-
	Proto. amoebophila	YP_007603	---------EGA--TN-LIHIY--	RKK	----IL--TFI---G-SSNSD-IEE-
	Sim. negevensis	TIGR	---------ESS--IN-LIY-YV-	RKK	----VL--SFI-T-G-SSDSD-IEE-
Verrucomicrobia/ Lentisphaerae	Bacterium Ellin514	ZP_02970829	---D----YEAQ--TS-L-Y-YL-	RKK	----FLT-TFF---G-GSDAD--K--
	Akkerman. muciniphila	YP_001877648	---D-----EVQ--TN-L-Y-YL-	RRR	----FL--TFM-Y-GFK-DRD-VSQ-
	Opitutaceae bacterium	ZP_02012834	---D--T--EVQ--IN-L-Y-YL-	RRR	----FLI-TLF---G-SDNTK--E--
	Victivallis vadensis	ZP_01924354	---D----M-VQ--IN-FIYIYL-	RRR	----FYI-TF---IG-P-NRD--AE
	Lentisphaera araneosa	NZ_ABCK01000017	---D----MEVQY-NH-LIYIFM-	RRR	----FLISTF---VYSESNYDL-
	Opitutus terrae PB90-1	YP_001817123	---D--T--EVQ--NN-L-Y-YL-	RRR	----FLI-TL---IG-SSDIDL-N--
	Verrucomicro. spinosum	ZP_02930291	---D--T--EVQ--TN-L-Y-YL-	RRR	----FL--TL--VIG-PGDED--K--
Planctomycetes	Rho. baltica	NP_866682	V--E----IEVNVTK--A-T----		QSG-FA--ML---MDPKYSTDA----
	K. stuttgartiensis	CAJ73710	---E----IEL-VGK--V-T----		QSG-----CF----SAEYANDE-II
	Bla. marina	EAQ82611	---E----IEINITK--SFT----		QSG-FA--TL---MSPELSDDAS-I-
Bacteroidetes-Chlorobi Group	Por. gingivalis	NP_904700	---FK---IE-AT-INNVMYAY--		-KK---V-TL---IGFEADKD---I-
	Bact. fragilis	YP_213594	---FK---IE-AT-INNVMYAY--		-KK---V-TL---IGFENDKD--EI-
	Cb. tepidum	AAM71403	-V-T----IE-QT-INNQI--Y--		QKKNFLV-AL---IGFAKDED--G--
Spirochetes	Tre. pallidum	NP_218681	---------E--I-Q-KDLIYAKL		KK-RILG-VF----H-E-REQ-IEA-
	Bor. burgdorferi	NP_212523	---------E--I-S-KDYLYVKI		-KKRILI-LF----GFD-REK-IET-
Cyanobacteria	Gloe. violaceus	NP_925229	L--N--A--K--T-AN-LVW----		KT---S-VVL-K--GLSDNE ---A-
	Nostoc sp. PCC 7120	NP_485634	L--N--A--K--T-RN-LVW----		KT---S-QVL-K--GLSDNE -F-A-
Deinococcus-Thermus	The. thermophilus	YP_005430	PL-K--P-I-L-VE-NGVVSMKVN		K--F-LVLL--V-G-DQ-TLRE---
	D. radiodurans	NP_294636	PM-K--P-IEL--AGDVLEMKVNK		--F-VSLL--V-GMDDAS-RA---
	T. maritima	NP_228268	FL-S--A--EVIL--Y-GVLYAGL		DGK-VNLFLF-KTIG-EKDED--S--
Actinobacteria	Myc. tuberculosis	ABK96916	V--S--A--E-DV-KR-TVG----		-K-RQ-V-VL-K--GW-S-- -VER-
	Cor. glutamicum	NP_599733	V--S--A--E-DV-KR-SVG----		-K-RQ-V-VL-K--GW---- -TER-
	Str. coelicolor	NP_628815	---S--A--EM-I-KR-MVG----		-K--QSV-VL-K--GW---- --EE-
	Bif. longum	AAN25011	---S--A--E--I-K--QPQ--V-		-K--QS-IVF-M-IGM-KSE -AQA-
Firmicutes	Bac. subtilis	NP_387988	V--N--A--EY-T-A--VVY----		-T----V-VL----GFGSD-E-----
	Sta. aureus	CAA45512	---N--A--EY-T-A--VVY----		-T----L-VL----GFSSD-E-V--L
	Strep. pneumoniae	NP_346388	V--N--A--EL-S-S--ITYT---		-T--I-F-TLV---GFSGDDE-F-I-
	Clo. perfringens	NP_563329	V--N--A--EY-T-SNSVIH----		-T----I–LV--MGFGSDAE -VNY-

Partial sequence alignment of the RpoB protein showing a 3 aa insert (boxed) that is uniquely found in all available *Chlamydiae* and *Verrucomicrobia-Lentisphaerae* groups of species, but not found in any other bacteria, including the *Planctomycetes* species. This insert was originally described in the Ref. 18 and its presence in various species from the *Chlamydiae* and *Verrucomicrobia-Lentisphaerae* phyla strongly indicates that species from these two phyla are most closely related to each other. The dashes in this alignment denote identity with the amino acid on the top line.
